# Malic enzyme 2 suppresses PINK1-Parkin-mediated mitophagy by stabilizing ATAD3A via competitive interaction with TRIM25

**DOI:** 10.1038/s41419-026-08623-2

**Published:** 2026-03-24

**Authors:** Qian Liu, Lei Su, Xiaoyun Wei, Shijie Lin, Lingkai Huang, Lige Hou, Yanhong Wang, Liubing Hu, Junyang Tan, Jing Qiao, Qinghua Zhou, Yi Ma, Wenjun Wang, Jianshuang Li

**Affiliations:** 1 State Key Laboratory of Bioactive Molecules and Druggability Assessment, Guangdong Basic Research Center of Excellence for Natural Bioactive Molecules and Discovery of Innovative Drugs, College of Life Science and Technology, Jinan University, Guangzhou, Guangdong China; 2 The First Affiliated Hospital, Key Laboratory of Regenerative Medicine of Ministry of Education, Jinan University, Guangzhou, Guangdong China; 3 Department of Orthopaedics, Guangzhou Red Cross Hospital, Faculty of Medical Science, Jinan University, Guangzhou, Guangdong China; 4 The Affiliated Qingyuan Hospital (Qingyuan People’s Hospital), Guangzhou Medical University, Qingyuan, Guangdong China

**Keywords:** Molecular biology, Cell signalling

## Abstract

Malic enzyme 2 (ME2), a pivotal enzyme related to the tricarboxylic acid (TCA) cycle, has been implicated in multiple cancers due to its overexpression and metabolic role in regulating the NADP^+^/NADPH balance. Malic enzyme 2 has been reported to regulate mitochondrial biogenesis and fusion; however, whether malic enzyme 2 participates in mitophagy regulation has remained unclear. Here, we reported that malic enzyme 2 depletion enhances PINK1-Parkin-mediated mitophagy. Mechanistically, ME2 competes with the E3 ubiquitin ligase TRIM25, disrupting its binding with ATPase family AAA domain-containing protein 3 A (ATAD3A), a mitochondrial protein crucial for the degradation of PINK1. Loss of malic enzyme 2 strengthens the TRIM25-ATAD3A interaction, resulting in ATAD3A ubiquitination and proteasomal degradation. The consequent PINK1 accumulation drives mitophagy activation. Hyperactivated mitophagy caused by malic enzyme 2 knockdown disrupts mitochondrial homeostasis, which suppresses the proliferative capacity of hepatoma cells. Moreover, pharmacological inhibition of mitophagy partially rescued the suppressed cell proliferation in the malic enzyme 2-knockdown cells. Our findings reveal a previously unrecognized role of malic enzyme 2 in mitochondrial quality control and highlight the ME2-ATAD3A-PINK1 axis as a potential regulatory node for mitophagy modulation.

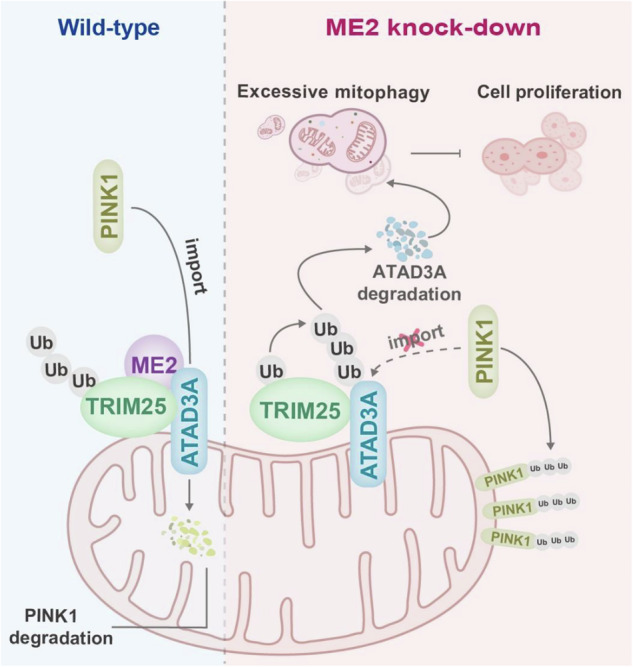

## Introduction

Mitophagy is a selective autophagic process that degrades damaged or dysfunctional mitochondria, thus preserving the balance between mitochondrial quality and quantity [1]. PINK1/Parkin mainly regulates ubiquitin-dependent mitophagy, which is crucial for many aspects of mitochondrial physiology, particularly the initiation of autophagic mechanisms [2]. The ATPase family AAA domain-containing protein 3 (ATAD3) is a transmembrane protein that is localized to both the inner and outer mitochondrial membranes. ATAD3 plays a crucial role in various cellular processes, including mitochondrial biogenesis, cholesterol transport, hormone synthesis, and apoptosis [3, 4]. ATAD3A plays a vital role in suppressing PINK1-dependent mitophagy. It facilitates the mitochondrial import and degradation of PINK1, thereby preventing excessive mitophagy activation. Loss or silencing of ATAD3A leads to PINK1 accumulation and subsequent mitophagy activation [5, 6].

The tricarboxylic acid (TCA) cycle, also known as the Krebs cycle, is a central mitochondrial metabolic pathway that serves as a crucial hub for cellular energy production and biosynthetic precursor generation [7, 8]. Recent studies have uncovered that TCA cycle-related enzymes are not only involved in classical metabolic functions but also regulate non-metabolic processes such as nuclear epigenetic modifications and influence DNA stability [9]. Aberrations in the TCA cycle are closely associated with a variety of pathological processes, including neurodegenerative diseases [10] and cancers [11, 12]. Notably, different TCA cycle-related enzymes possess unique biological functions, and their deficiencies or dysfunctions lead to specific pathological phenotypes [13]. However, the role of TCA cycle-related enzymes in regulating the mitochondrial quality control through non-metabolic mechanisms remains unclear.

Malic enzyme 2 is a mitochondrial NADP^+^-dependent enzyme that catalyzes the conversion of malate to pyruvate, playing a pivotal role in the TCA cycle and energy metabolism [14, 15]. It functionally couples mitochondrial respiration with aerobic glycolysis in processes like osteoblast differentiation, where its knockdown impairs glycolytic flux and cellular proliferation [16]. Malic enzyme 2 also regulates mitochondrial biomass production by sensing nutrient availability through fumarate binding, which promotes dimerization and activates downstream pathways for mtDNA synthesis and mitoribosome assembly [17]. Additionally, malic enzyme 2 is critical for odontoblastic differentiation and mitochondrial fusion, with its spatiotemporal expression in tooth germs linking melatonin to mitochondrial respiration [18]. Malic enzyme 2 is a key mitochondrial enzyme that contributes to tricarboxylic acid cycle–linked metabolism and supports mitochondrial homeostasis. However, the potential non-canonical roles and precise mechanisms by which malic enzyme 2 regulates PINK1-Parkin-mediated mitophagy and mitochondrial quality control signaling pathways remain to be fully elucidated.

Here, we identify malic enzyme 2 as a negative regulator of PINK1-Parkin-mediated mitophagy. In wild-type cells, malic enzyme 2 competes with TRIM25 for ATAD3A binding, preventing TRIM25-mediated ubiquitination and degradation of ATAD3A. Malic enzyme 2 deficiency enhances TRIM25-ATAD3A interaction, accelerates ATAD3A degradation, and triggers excessive mitophagy, impairing mitochondrial function and inhibiting hepatocellular carcinoma (HCC) cell proliferation. Pharmacological mitophagy inhibition with chloroquine or Mdivi-1 rescues the growth defect caused by malic enzyme 2 knockdown. These findings uncover a ME2-ATAD3A-PINK1 axis in mitochondrial quality control and suggest that targeting this pathway may have therapeutic potential in liver cancer.

## Results

### Knockdown of malic enzyme 2 gene promotes PINK1-Parkin-mediated mitophagy

**Fig. 1:**
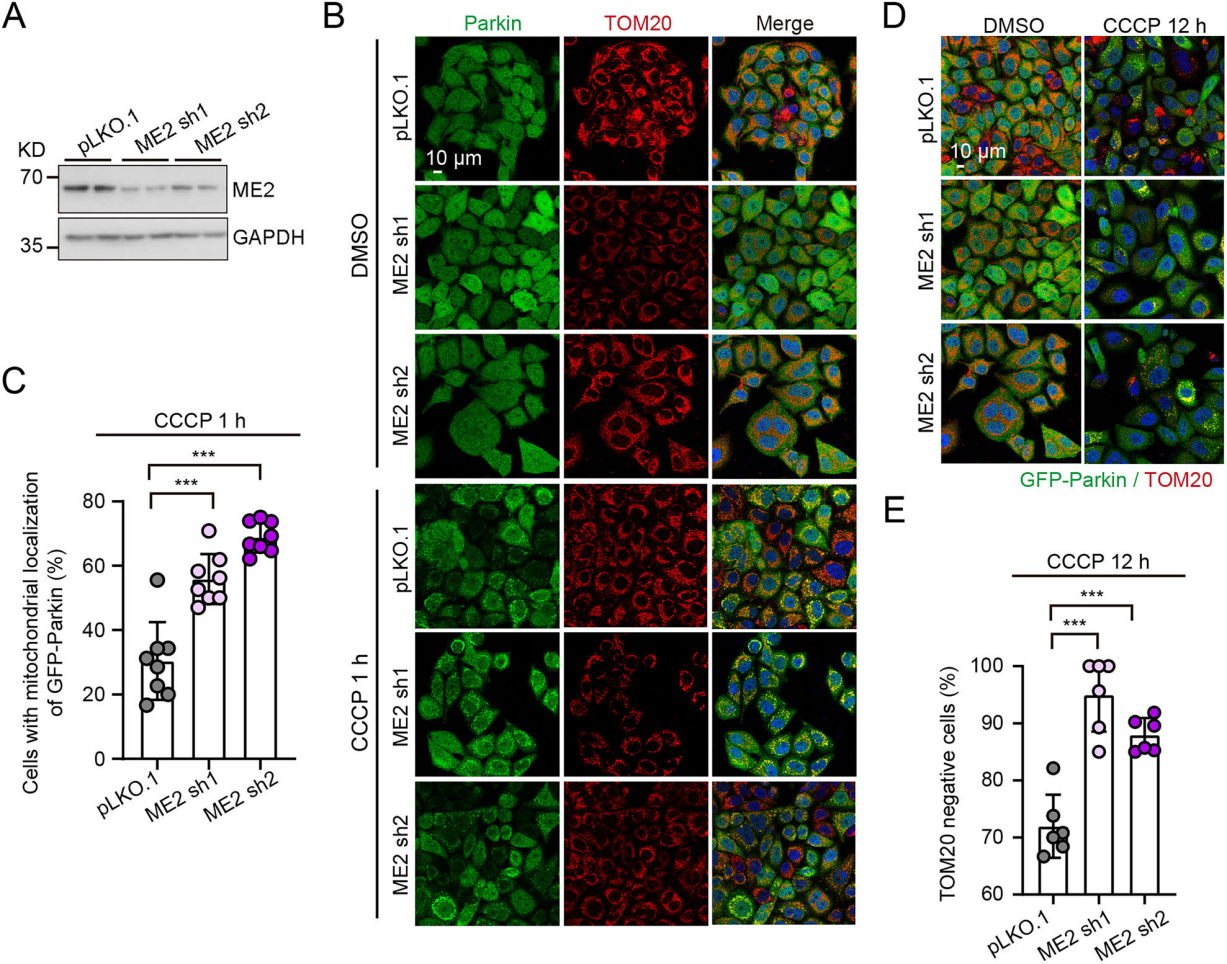
Suppression of malic enzyme 2 (ME2) promotes mitophagy in GFP-Parkin HeLa cells. **A** Western blot analysis of ME2 and GAPDH protein levels in control (pLKO.1) and ME2 knockdown GFP-Parkin HeLa cells. Representative immunofluorescence images (**B**) and quantitative analysis (**C**) of the Parkin and TOM20 colocalization in pLKO.1 and ME2 knockdown GFP-Parkin HeLa cells with 10 μM CCCP treatment for 1 hour. Scale bar, 10 μm. *n* = 8. Representative images (**D**) and quantification (**E**) of TOM20-negative cells with 10 μM CCCP treatment for 12 hours. Scale bar, 10 μm. *n* = 6. ***p < 0.001.

To study the role of metabolism-related enzymes in regulating mitophagy, we screen a shRNA library in GFP-Parkin-expressing HeLa cells, a well-established mitophagy model. We observed that malic enzyme 2 knockdown markedly increased CCCP-induced GFP-Parkin puncta formation, suggesting enhanced Parkin recruitment to mitochondria. Malic enzyme 2, a mitochondrial TCA cycle-related enzyme involved in mitochondrial biogenesis, dynamics, and mitochondrial respiratory function [17, 18]. However, its role in mitophagy regulation remains unexplored. We further confirmed that malic enzyme 2 knockdown significantly increased the percentage of cells in which GFP-Parkin recruitment to mitochondria compared to control pLKO.1 cells (Fig. 1A–C). Extended CCCP treatment for 12 hours increased the proportion of TOM20-negative cells from ~70% in controls to ~90% in malic enzyme 2-depleted cells (Fig. 1D, E), indicating a heightened mitophagy response.

**Fig. 2:**
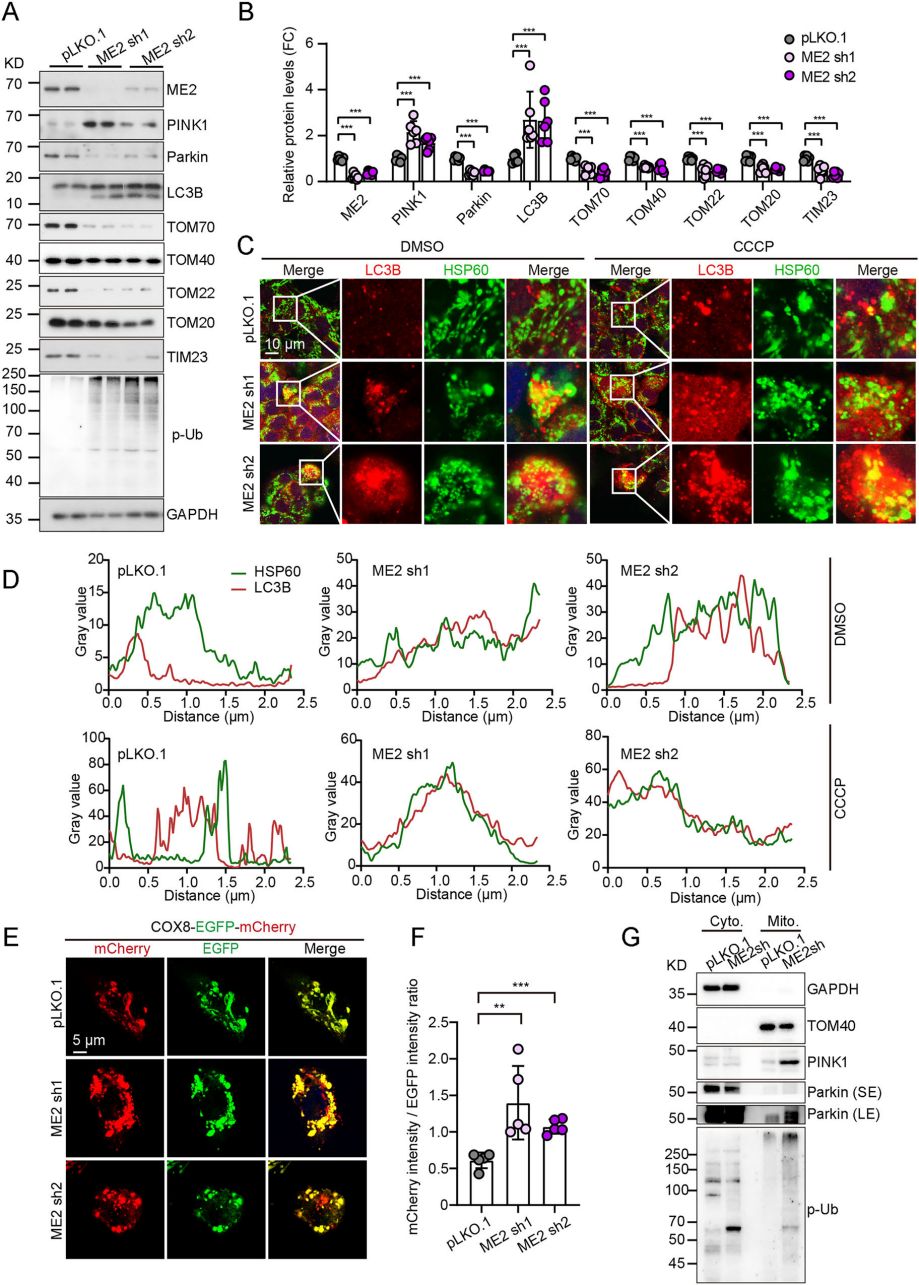
Malic enzyme 2(ME2) Deficiency promotes PINK1-Parkin-mediated mitophagy in HepG2 cells. Western blot analysis (**A**) and quantification (**B**) of mitophagy-related proteins in control (pLKO.1) and ME2-knockdown HepG2 cells. *n* = 6. Representative images (**C**) and quantification (**D**) of LC3B and HSP60 colocalization in pLKO.1 and ME2 knockdown HepG2 cells under normal and CCCP treatment conditions. CCCP: 10 μM for 6 hours. Scale bar, 10 μm. Representative images (**E**) and quantification (**F**) of mitophagic flux in pLKO.1 and ME2 knockdown HepG2 cells transfected with the mitophagy reporter COX8-EGFP-mCherry for 48 hours. Scale bar, 5 μm. *n* = 5. **G** Immunoblot analysis of phosphorylated ubiquitin (p-Ub), PINK1, and Parkin in cytosolic and mitochondrial fractions. GAPDH and TOM40 serve as markers for cytosolic and mitochondrial fractions, respectively. **p < 0.01, ***p < 0.001.

Given that malic enzyme 2 was originally identified in hepatoma mitochondria [15], we next investigated its role in HepG2 cells. Malic enzyme 2 knockdown enhanced the accumulation of PINK1 and the degradation of Parkin (Fig. 2A, B), indicating activation of the PINK1-Parkin pathway. Moreover, the lipidation of LC3B dramatically increased in malic enzyme 2 knockdown cells, concurrent with reduced levels of mitochondrial proteins, including TOM70, TOM40, TOM22, TOM20, and TIM23 (Fig. 2A, B).

Furthermore, co-staining of HSP60 (a mitochondrial marker) and LC3B (an autophagosome marker) increased in malic enzyme 2 knockdown cells both at baseline and after CCCP treatment (Fig. 2C, D). Additionally, using the COX8-EGFP-mCherry mitophagy reporter, malic enzyme 2 knockdown caused a shift from yellow to red-only puncta and increased the mCherry/EGFP ratio (Fig. 2E, F). Moreover, subcellular fractionation showed increased mitochondrial PINK1, Parkin, and phosphorylated ubiquitin in malic enzyme 2knockdown cells (Fig. 2G). Conversely, malic enzyme 2 overexpression reduced mitochondrial Parkin recruitment (Fig. S1), decreased LAMP1-mitochondria colocalization (Fig. S2A). Meanwhile, malic enzyme 2 overexpression suppressed PINK1-Parkin activation and restored mitochondrial protein levels upon CCCP treatment (Fig. S2B). Collectively, these results demonstrate that malic enzyme 2 suppresses the PINK1-Parkin-mediated mitophagy.

### Loss of malic enzyme 2 induces mitochondrial dysfunction and suppresses cell proliferation

**Fig. 3:**
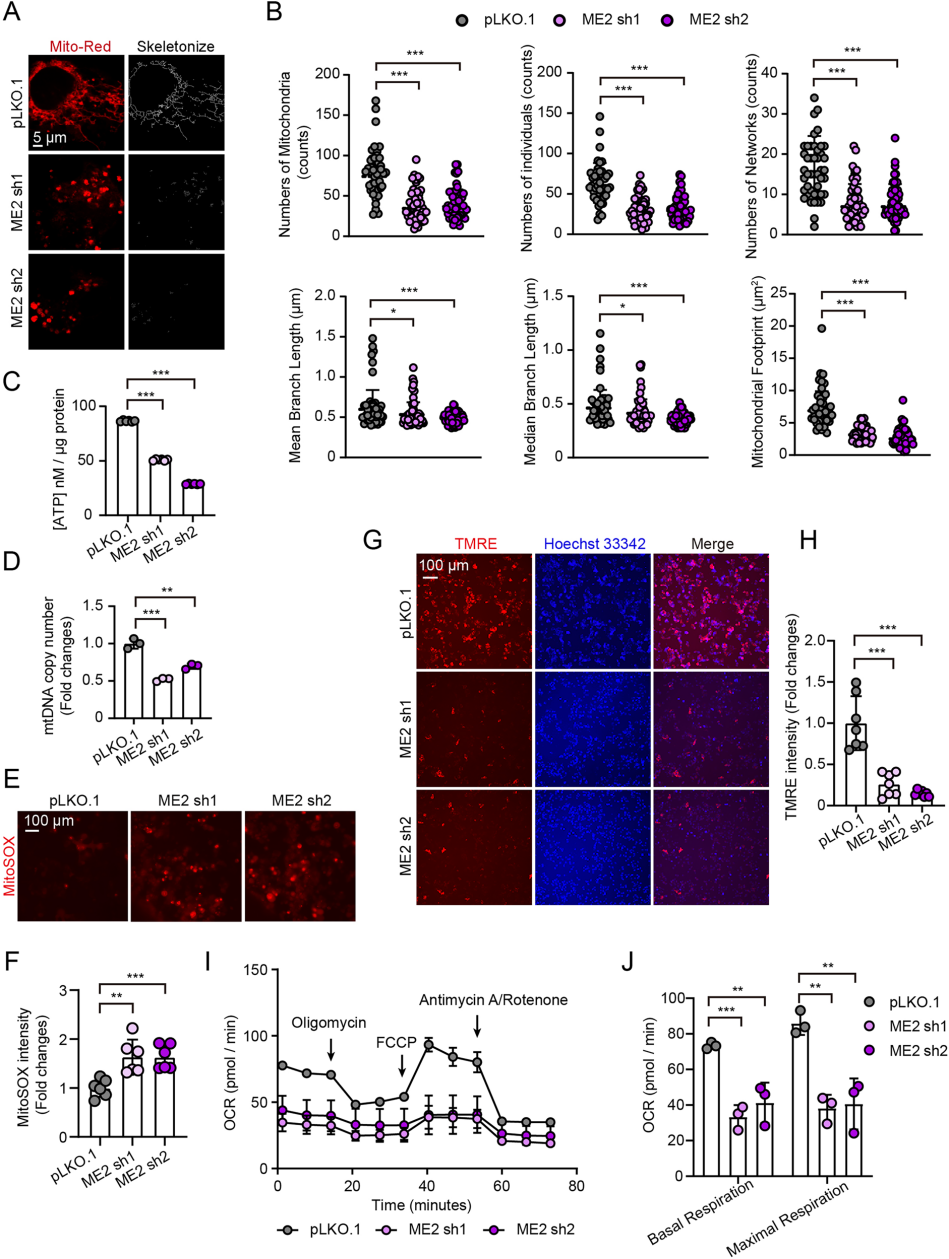
Malic enzyme 2 (ME2) deficiency induces mitochondrial morphological abnormalities and impairs mitochondrial function. **A, B** Mitochondrial morphology visualized by MitoTracker Red staining in control and ME2-knockdown HepG2 cells. Mitochondrial networks were skeletonized and quantified using the Mitochondrial Network Analysis (MiNA) tool. *n* = 50. Scale bar, 5 μm. **C** ATP levels. *n* = 6. **D** Mitochondrial DNA copy number. *n* = 3. **E, F** Mitochondrial reactive oxygen species (ROS) levels. Scale bar, 100 μm. *n* = 6. Representative confocal images (**G**) and quantification (**H**) of TMRE staining. Scale bar, 100 μm. *n* = 7. **I–J** Oxygen consumption rate (OCR) analysis in pLKO.1 and ME2 knockdown HepG2 cells. *n* = 3. *p < 0.05, **p < 0.01, ***p < 0.001.

Mitophagy is a selective autophagy that removes damaged mitochondria to maintain mitochondrial quality and cell homeostasis. However, excessive mitophagy can impair mitochondrial function and repress cell proliferation [19–21]. Next, we examined mitochondrial morphology and function in malic enzyme 2-deficient HepG2 cells. MitoTracker Red staining revealed pronounced mitochondrial fragmentation after malic enzyme 2 knockdown (Fig. 3A). Quantitative analysis of mitochondrial networks showed that the knockdown of malic enzyme 2 significantly decreased the mitochondrial number, connectivity, and branch length (Fig. 3B). Additionally, loss of malic enzyme 2 reduced ATP production and mitochondrial DNA (mtDNA) copy number (Fig. 3C, D). Mito-SOX and TMRE staining demonstrated increased mitochondrial reactive oxygen species (ROS) and decreased mitochondrial membrane potential (ΔΨm) upon malic enzyme 2 loss (Fig. 3E–H). Seahorse analysis showed that basal and maximal oxygen consumption rates (OCR) were significantly decreased in malic enzyme 2-deficient cells (Fig. 3I, J). While malic enzyme 2 overexpression enhanced the basal and maximal respiration (Figure S2C, D), ATP production, and mtDNA copy number (Fig. S2E, F). Furthermore, malic enzyme 2 knockdown in HepG2 cells significantly inhibited cell proliferation, colony formation, and sphere formation (Fig. S3A–E), suggesting reduced self-renewal capacity. Given that malic enzyme 2 depletion leads to hyperactivated mitophagy and suppressed cell proliferation, we next asked whether inhibiting mitophagy could reverse these effects. Treatment with chloroquine (CQ) or Mdivi-1 (an inhibitor of PINK1-Parkin mediated mitophagy) partially restored the levels of mitochondrial outer membrane proteins (TOM70 and TOM40) and phosphorylated ubiquitin (p-Ub) in ME2-knockdown cells (Fig. S3F, G), indicating effective suppression of ME2-induced mitophagy. Furthermore, CQ and Mdivi-1 partially rescued the proliferation defect observed in malic enzyme 2-deficient HepG2 cells (Fig. S3H, I). Because malic enzyme 2 knockdown enhances PINK1-Parkin dependent mitophagy, we also disrupted this pathway using two independent PINK1-specific siRNAs. Silencing PINK1 reversed the anti-proliferative effect caused by ME2 knockdown (Fig. S3J, K), supporting the conclusion that ME2 loss impairs cell proliferation through mitophagy activation. Collectively, these findings indicate that malic enzyme 2 deficiency disrupts mitochondrial morphology and compromises respiratory function, ultimately leading to suppressed cell proliferation via excessive mitophagy.

### Malic enzyme 2 suppresses mitophagy in a manner independent of its enzymatic activity

**Fig. 4:**
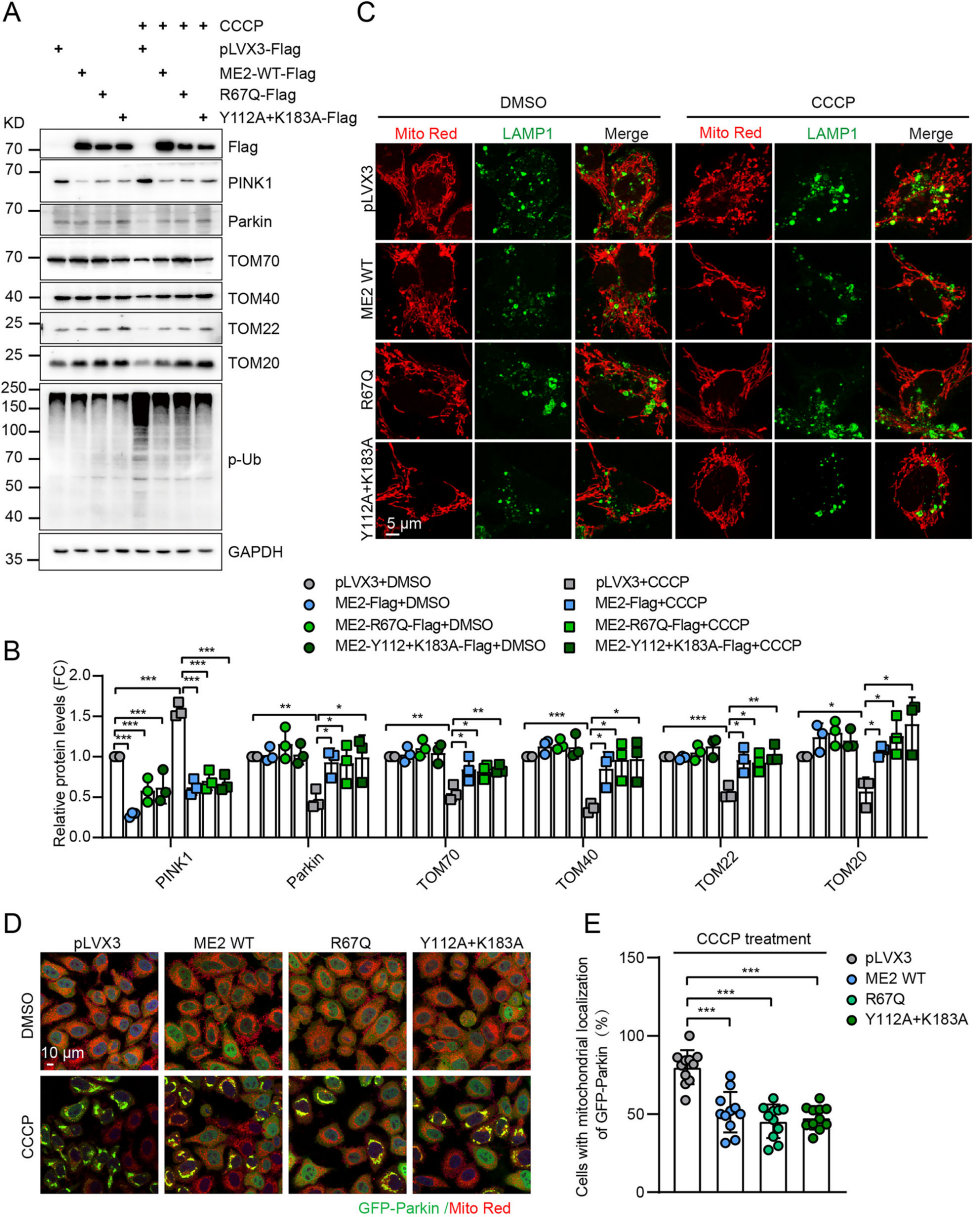
Malic enzyme 2(ME2) regulates mitophagy independent of its enzymatic activity. Western blot analysis (**A**) and quantification (**B**) of mitophagy-associated proteins in HepG2 cells overexpressing empty vector (pLVX3), wild-type ME2 (WT), or enzymatically deficient ME2 mutants (R67Q, Y112A + K183A). **C** Representative images of colocalization of the LAMP1 with mitochondria. Scale bar, 5 μm. Representative images (**D**) and quantification (**E**) of Parkin and mitochondrial colocalization in cells overexpressing pLVX3, WT or mutant (R67Q, Y112A + K183A) ME2. Scale bar, 10 μm, *n* = 11. *p < 0.05, **p < 0.01, ***p < 0.001.

Since malic enzyme 2 is a metabolic enzyme that catalyzes the oxidative decarboxylation of L-malate into pyruvate, mutations affecting the catalytic activity of malic enzyme 2 may induce alterations in structural properties, thereby affecting downstream functions. Next, we investigated whether its enzymatic activity is required for mitophagy regulation. We generated two catalytically deficient malic enzyme 2 mutants (R67Q, Y112A + K183A) [22–24]. Both wild-type malic enzyme 2 and catalytically inactive mutants (R67Q, Y112A + K183A) suppressed CCCP-induced mitochondrial protein degradation, including TOM20, TOM22, TOM40, TOM70, as well as PINK1-Parkin activation (Fig. 4A, B). Additionally, all malic enzyme 2 variants reduced CCCP-induced LAMP1/Parkin - mitochondria colocalization and decreased the percentage of cells exhibiting with GFP-Parkin recruitment to mitochondria (Fig. 4C-E). These results indicate that malic enzyme 2 regulates mitophagy independently of its enzymatic activity.

### Malic enzyme 2 inhibits ATAD3A proteasomal degradation by competitive interaction with TRIM25

**Fig. 5:**
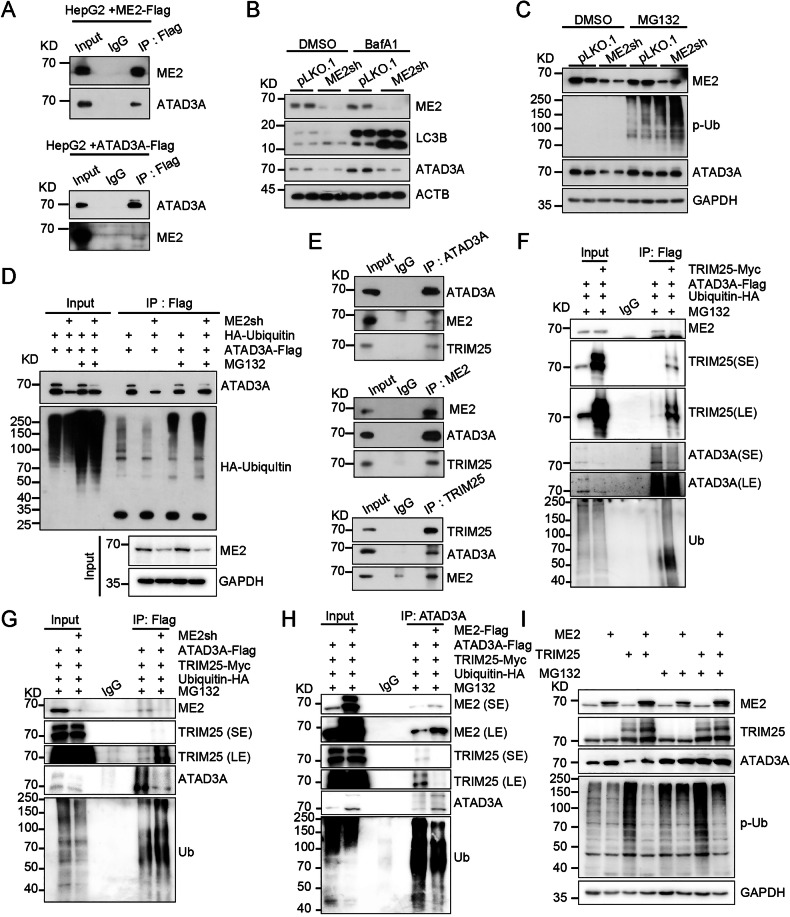
Malic enzyme 2 (ME2) stabilizes ATAD3A by inhibiting TRIM25-mediated ubiquitination. **A** Co-immunoprecipitation (Co-IP) validating the interaction between ME2 and ATAD3A. HepG2 cells were transfected with ME2-Flag or ATAD3A-Flag plasmids for 48 hours. **B, C** Western blot analysis of ATAD3A in pLKO.1 and ME2 knockdown HepG2 cells upon 100 nM BafA1 or 10 μM MG132 treatment for 8 hours. **D** Co-IP analyzed the ubiquitination of ATAD3A. The pLKO.1 and ME2 knockdown 293 T cells were co-transfected with ATAD3A-Flag and HA-ubiquitin (HA-Ub) plasmids for 48 hours and then treated with 10 μM MG132 for 8 hours. **E** Endogenous interaction among ME2, ATAD3A, and TRIM25 assessed by Co-IP in HepG2 cells. **F** Co-IP analyzed TRIM25 binds to and ubiquitinates ATAD3A. 293 T cells were co-transfected with the indicated plasmids with 48 hours and then treated with 10 μM MG132 for another 8 hours. **G, H** Co-IP analysis of TRIM25-ATAD3A interaction and ubiquitination of ATAD3A in the presence or absence of ME2. pLKO.1 and ME2- knockdown 293 T cells were co-transfected with ATAD3A-Flag, TRIM25-Myc and Ubiquitin-HA (**G**). 293 T cells were co-transfected with ATAD3A-Flag, TRIM25-Myc and Ubiquitin-HA, and with or without ME2-Flag (**H**). Following 48 hour of transfection, cells were treated with 10 μM MG132 for another 8 hours and harvested for Co-IP analysis. **I** Western blot analysis of ATAD3A levels. HepG2 cells were co- or alone transfected with ME2 and TRIM25 for 48 hours and then treated within 10 μM MG132 for another 8 hours.

To elucidate how malic enzyme 2 inhibits mitophagy, we performed co-immunoprecipitation (Co-IP) followed by mass spectrometry to identify potential malic enzyme 2 interactors involved in mitophagy. Among the identified candidates, we focused on ATAD3A (Fig. S4A), a mitochondrial transmembrane protein known to be involved in mitochondrial biogenesis and mitophagy [25]. We confirmed the interaction between malic enzyme 2 and ATAD3A via Co-IP (Fig. 5A). Notably, malic enzyme 2 overexpression increased ATAD3A protein abundance, whereas malic enzyme 2 knockdown reduced ATAD3A protein without affecting mRNA levels (Fig. S4B–G), indicating malic enzyme 2 regulates ATAD3A through post-translational regulation. Considering that proteasomal and autophagic degradation are key mechanisms for protein turnover, we treated pLKO.1 and malic enzyme 2- knockdown cells with the autophagy inhibitor bafilomycin A1 (BafA1) and the proteasome inhibitor MG132. We found that treatment with BafA1 did not prevent ATAD3A reduction in malic enzyme 2-knockdown cells, whereas proteasome inhibitor MG132 restored ATAD3A’s expression (Fig. 5B, C, and S5), suggesting that malic enzyme 2 knockdown suppresses ATAD3A expression through proteasomal degradation. Furthermore, the Co-IP analysis revealed that malic enzyme 2 knockdown enhanced the ubiquitination of ATAD3A, which contributes to the degradation of ATAD3A (Fig. 5D). ATAD3A had been reported to play a role in the PINK1-Parkin-dependent mitophagy [26]. Consistently, we also found that ATAD3A knockdown promoted the accumulation of PINK1 and the degradation of Parkin (Fig. S6). Additionally, mitochondrial proteins such as TOM70, TOM40, TOM22, and TOM20 were decreased in ATAD3A- knockdown cells, accompanied by increased LC3B lipidation, a hallmark of autophagy (Fig. S6). These data suggest that malic enzyme 2 knockdown promotes mitophagy through enhancing the proteasomal degradation of ATAD3A.

We next sought to identify the E3 ubiquitin ligase mediating ATAD3A degradation in malic enzyme 2-deficient cells. Integrating predicted E3 ligases for ATAD3A and malic enzyme 2 pull-down candidate identified TRIM25 as a likely regulator (Fig. S7). Endogenous Co-IP assays confirmed the presence of a complex containing malic enzyme 2, ATAD3A, and TRIM25 (Fig. 5E). Further experiments showed that TRIM25 directly binds ATAD3A and promotes its ubiquitination and subsequent degradation (Fig. 5F). These results suggested that malic enzyme 2 could influence ATAD3A expression through protein-protein interactions.

To test whether malic enzyme 2 modulates ATAD3A stability via TRIM25-mediated ubiquitination, we performed co-immunoprecipitation experiments. Malic enzyme 2 knockdown enhanced the interaction between TRIM25 and ATAD3A, which was accompanied by increased ubiquitination and degradation of ATAD3A (Fig. 5G). Conversely, malic enzyme 2 overexpression weakened the TRIM25-ATAD3A interaction and reduced ATAD3A ubiquitination and degradation (Fig. 5H). Moreover, inhibition of the ubiquitin-proteasome pathway with MG132 blocked TRIM25-mediated degradation of ATAD3A (Fig. 5I). Together, these findings suggest that malic enzyme 2 stabilizes ATAD3A by competitively binding to TRIM25, thereby shielding ATAD3A from TRIM25-mediated ubiquitination and proteasomal degradation.

### Malic enzyme 2 overexpression rescues TRIM25-mediated mitophagy

**Fig. 6:**
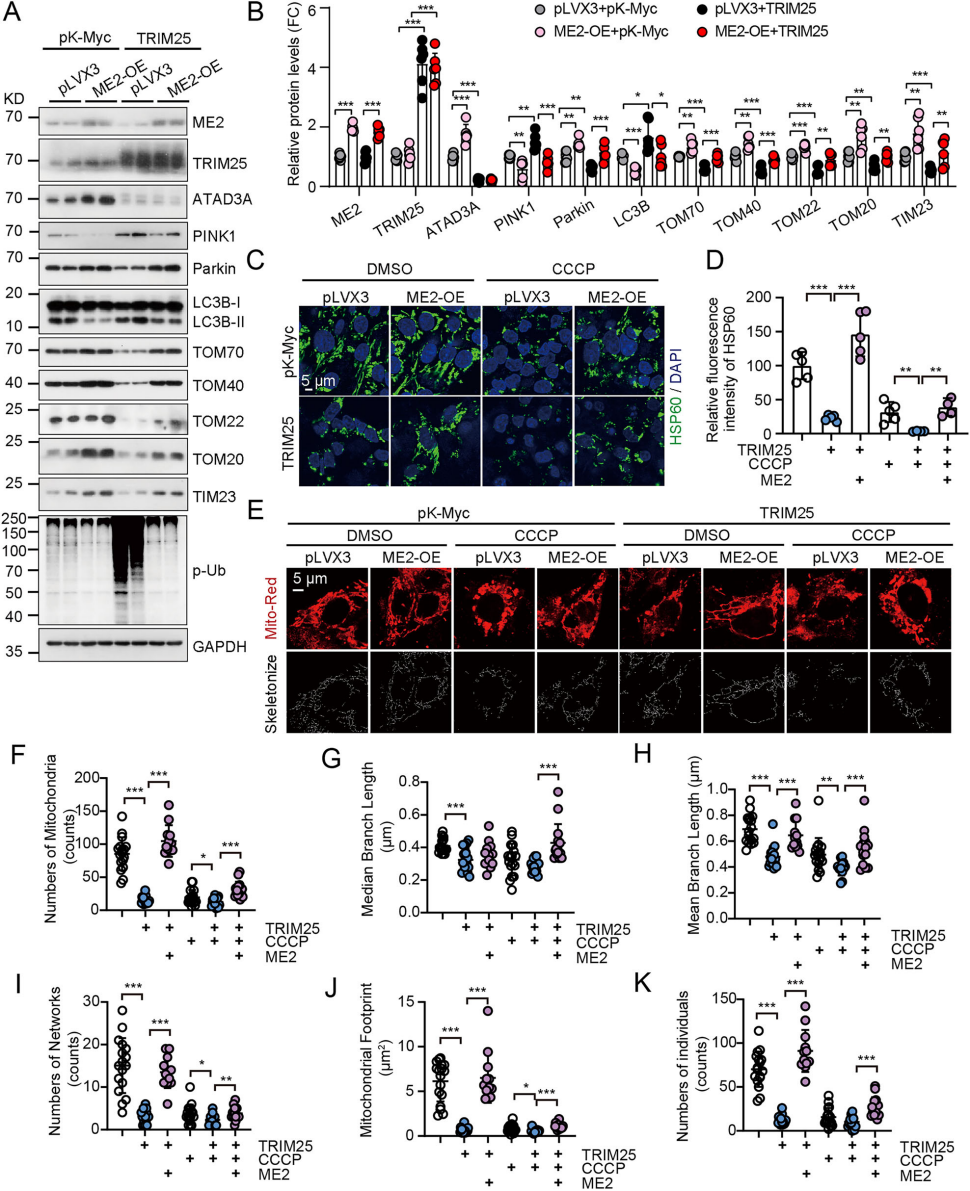
Malic enzyme 2 (ME2) overexpression attenuates TRIM25-mediated mitochondrial damage. Western blots (**A**) and quantification (**B**) of the indicated mitophagy-related protein. The control and ME2-overexpressed HepG2 cells were transfected with pK-Myc or TRIM25 for 48 hours. Representative images (**C**) and quantification (**D**) of HSP60 degradation in control and ME2-overexpressed HepG2 cells transfected with or without TRIM25 following 10 μM CCCP treatment for 6 hours. Scale bar, 5 μm, *n* = 5. Mitochondrial morphology analysis via MitoTracker Red staining (**E**) and corresponding quantitative metrics (**F**–**K**) in the indicated cell groups treated with 10 μM CCCP for 6 hours. Mitochondrial networks were skeletonized and quantified using the MiNA tool. Scale bar, 5 μm, *n* = 17. *p < 0.05, **p < 0.01, ***p < 0.001.

**Fig. 7:**
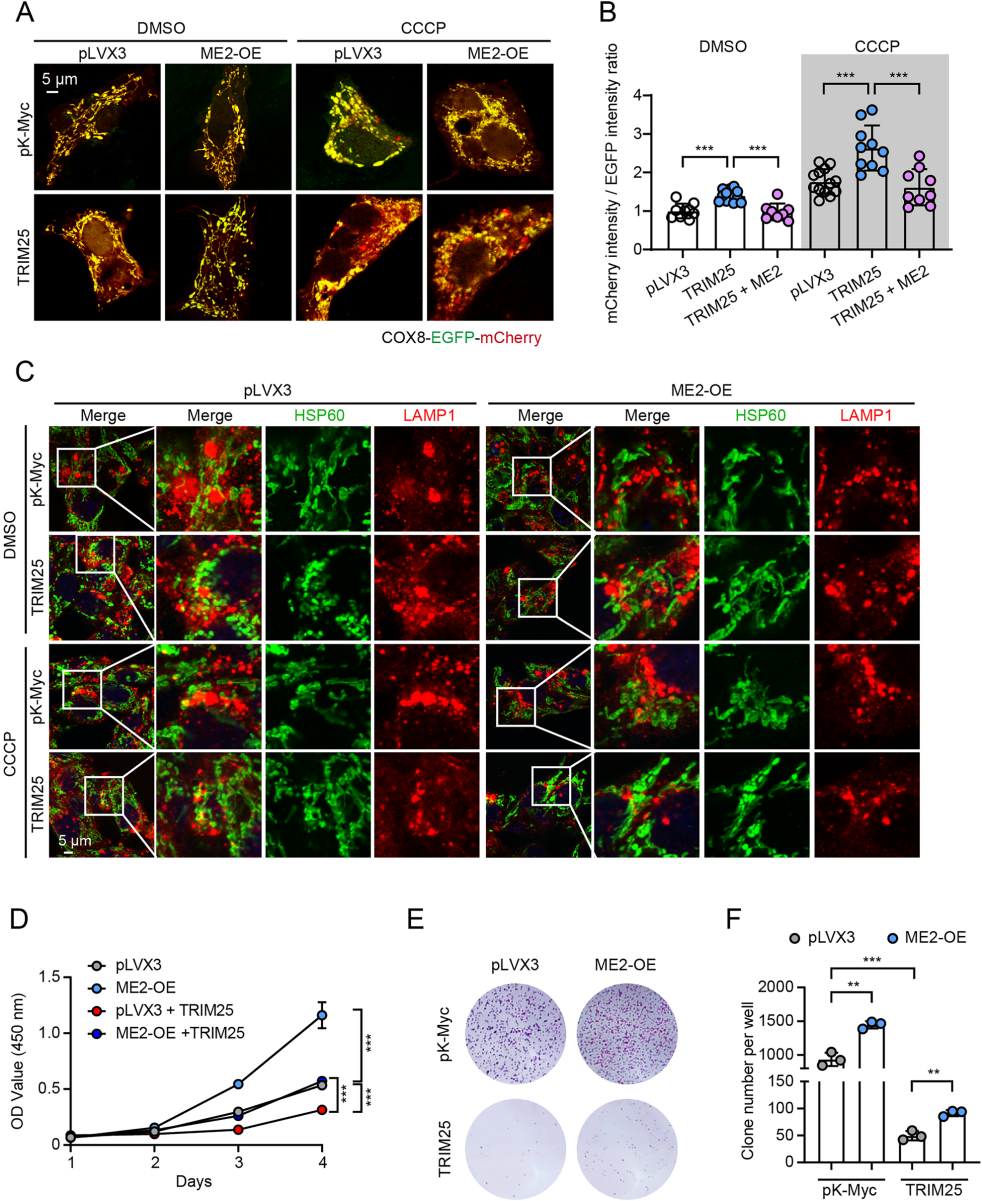
Malic enzyme 2 (ME2) rescues TRIM25-mediated mitophagy and cell proliferation suppression. Representative images (**A**) and quantification (**B**) of mitophagic flux in control and ME2-overexpressed HepG2 cells transfected with or without TRIM25. Cells were transfected with COX8-EGFP-mCherry following 10 μM CCCP treatment for 6 hours. Scale bar, 5 μm, *n* = 8–13. **C** Representative images showing the colocalization of the LAMP1 with HSP60 in control and ME2-overexpressed HepG2 cells transfected with or without TRIM25 following 10 μM CCCP treatment for 6 hours. Scale bar, 5 μm. **D** Cell viability measured by CCK-8 assay in control and ME2-overexpressed HepG2 cells with or without TRIM25 expression. Representative images (**E**) and quantification (**F**) of colony formation in control and ME2-overexpressed HepG2 cells with or without TRIM25 expression. *n* = 3. **p < 0.01, ***p < 0.001.

Next, we wonder whether TRIM25-mediated ATAD3A degradation causes mitophagy and whether it can be restored by malic enzyme 2 overexpression. Consistently, TRIM25 overexpression decreased ATAD3A protein levels, promoted PINK1-Parkin pathway activation, and decreased the abundance of mitochondrial proteins. Conversely, malic enzyme 2 overexpression suppressed TRIM25-induced mitophagy, as reflected by the restored protein levels of TOM70, TOM40, TOM22, and TOM20 levels under both basal and CCCP-treated conditions (Fig. 6A, B, Fig. S8A, B). Furthermore, malic enzyme 2 overexpression also blocked HSP60 degradation induced by TRIM25 overexpression both in normal and CCCP treatment conditions (Fig. 6C, D), and restored mitochondrial morphology (Fig. 6E–K). Moreover, under both the normal and CCCP treatment conditions, malic enzyme 2 overexpression abrogated TRIM25-induced mitophagy (Fig. 7A, B) and colocalization of LAMP1 or Parkin with mitochondria (Fig. 7C, Fig. S8C, D). We next examined whether TRIM25 also regulates cell proliferation, and malic enzyme 2 could restore it. Consistently, ME2 overexpression could restore TRIM25 overexpression-mediated suppression of cell proliferation (Fig. 7D–F), indicating that malic enzyme 2 promotes growth, at least in part, by antagonizing TRIM25 activity and suppressing mitophagy. These findings demonstrate that malic enzyme 2 competitively interacts with TRIM25, thereby protecting ATAD3A from proteasomal degradation and repressing PINK1-Parkin-mediated mitophagy.

Together, these findings define malic enzyme 2 as a negative regulator of PINK1-Parkin-mediated mitophagy. By competitively binding to TRIM25, malic enzyme 2 prevents TRIM25-dependent ubiquitination and proteasomal degradation of ATAD3A, thereby maintaining PINK1 turnover and restraining mitophagy under basal and stress conditions. Loss of malic enzyme 2 disrupts this protective interaction, leading to ATAD3A depletion, PINK1 accumulation on the outer mitochondrial membrane, which induces hyperactivated mitophagy. This excessive mitochondrial clearance impairs mitochondrial morphology, bioenergetics, and redox balance, ultimately suppressing cell proliferation. These results uncover a previously unrecognized ME2-TRIM25-ATAD3A-PINK1 axis that links mitochondrial metabolic enzymes to mitochondrial quality control.

## Discussion

In this study, we demonstrated that loss of malic enzyme 2 leads to hyperactivation of PINK1-Parkin-dependent mitophagy, resulting in impaired mitochondrial function and reduced cell proliferation. Mechanistically, we found that malic enzyme 2 competitively interacts with the E3 ubiquitin ligase TRIM25, preventing TRIM25 from interacting with ATAD3A, a mitochondrial transmembrane protein that facilitates PINK1 import into mitochondria for degradation. In malic enzyme 2-deficient cells, enhanced TRIM25-ATAD3A interaction promotes ATAD3A ubiquitination and proteasomal degradation, which prevents PINK1 import and leads to its accumulation on the outer mitochondrial membrane, thereby activating the PINK1-Parkin pathway. Overexpression of malic enzyme 2, or its catalytically inactive mutants, suppressed mitophagy, and pharmacological inhibition of mitophagy restored the proliferation defect caused by malic enzyme 2 depletion, indicating that this role of malic enzyme is independent of its catalytic activity.

Elevated expression of malic enzyme 2 has been reported in multiple human cancers, including colorectal cancer [27], acute myeloid leukemia cells [28], hepatocellular carcinoma cells [29], lung cancer cells [30], and breast cancer cells [31]. Loss of malic enzyme 2 has been shown to suppress the proliferation and migration of cancer cells across various cell lines. While impaired mitophagy has been linked to cancer progression, we found that ME2 depletion inhibits HepG2 cell proliferation through hyperactivation of mitophagy (Fig. S3). However, the underlying mechanism by which ME2 regulates liver cancer via the ATAD3A-PINK1-mitophagy axis remains to be fully elucidated and requires further investigation. In many cancer models, malic enzyme 2 depletion suppresses cell growth by altering ROS, ATP, and NADP⁺/NADPH ratios in a manner dependent on its enzymatic activity [28, 30, 32]. Our results focus on a distinct mechanism - mitochondrial quality control via mitophagy. Similar to a recent report showing that malic enzyme 2 deficiency impairs mitochondrial respiration [27], we found that malic enzyme 2 knockdown reduced mitochondrial respiratory capacity, increased ROS, and caused fragmentation of mitochondrial networks (Fig. 3). A previous study also linked malic enzyme 2 to mitochondrial fusion in rat dental papilla cells, which promoted tooth development [18]. In line with this, our mitochondrial morphology analysis revealed that loss of malic enzyme 2 not only caused mitochondrial fragmentation but also reduced the mitochondrial mass (Fig. 3A–D). Interestingly, a previous study reported that malic enzyme 2 regulated mitochondrial biogenesis by binding with fumarate [17]. In this study, Wang et al. demonstrated that the Krebs cycle intermediate fumarate binds with malic enzyme 2, which promoted the formation of the malic enzyme 2 dimer. On one hand, the malic enzyme 2 dimer activated nucleotidohydrolase (DUT) for mtDNA synthesis; on the other hand, formation of the malic enzyme 2 dimer inhibited the malic enzyme 2 monomer from binding with mitochondrial ribosome protein L45, thereby freeing it for mitochondrial biogenesis [17]. These data revealed a novel function of malic enzyme 2 in mitochondrial biogenesis. Our data found another role of malic enzyme 2 in mitochondrial quality control through PINK1-Parkin-dependent mitophagy (Figs. 1, 2). However, malic enzyme 2 knockdown may extend beyond simply reducing enzymatic activity and could broadly disrupt mitochondrial integrity, including protein import machinery. This widespread disruption of mitochondrial homeostasis, such as impaired protein import or structural instability, may indirectly drive the observed hyperactivation of mitophagy, a mechanism requiring further investigation.

Mitophagy plays context-dependent roles in cancer, functioning either to support tumor growth by removing damaged mitochondria or to suppress tumor progression under certain conditions. Impaired mitophagy can exacerbate ROS accumulation and activate inflammatory signaling, as shown in breast cancer models where ULK1 or BRCA1 deficiency promoted metastasis via NLRP3 inflammasome activation [19, 20]. Conversely, excessive mitophagy may reduce mitochondrial biomass and ATP production, limiting the proliferative capacity of high-energy-demanding cancer cells. Our findings support the latter scenario: Malic enzyme 2 depletion induced hyperactivated mitophagy, which correlated with impaired proliferation in HepG2 cells. A recent study demonstrated that deletion of ATAD3A hyperactivated mitophagy in mouse hematopoietic cells, which caused severely decreased bone-marrow cellularity, erythroid anemia, and B cell lymphopenia, and finally reduced mice survival [5]. Consistent with their work, we also found that knockdown of ATAD3A promoted PINK1 accumulation, decreased mitochondrial proteins (Fig. S6), indicating that hyperactivated mitophagy contributed to cell growth inhibition or decreased cell viability. Furthermore, we found that the knockdown of malic enzyme 2 repressed ATAD3A at the protein level but not the mRNA level (Fig. S4B–G). Knockdown of malic enzyme 2 enhanced the interaction between ATAD3A and TRIM25, which leads to the ubiquitination of ATAD3A and the subsequent degradation (Fig. 5G). Jin et al. demonstrated that ATAD3A interacted with both the mitochondrial outer membrane channel TOM40 and the inner channel TIM23, and served as a bridging factor for PINK1’s transportation into mitochondria and the subsequent degradation [5]. Here, we also found that the knockdown of malic enzyme 2 caused the accumulation of PINK1 (Fig. 2A). These data demonstrated that deficiency of malic enzyme 2 or ATAD3A mediated hyperactivated PINK1-Parkin-dependent mitophagy.

In Fig. 6A, our data demonstrated that the overexpression of malic enzyme 2 inhibited TRIM25-mediated mitophagy without restoring ATAD3A expression. We propose that this excess of TRIM25 likely drove extensive ubiquitination and degradation of ATAD3A, which may explain why the protective effect of ME2 overexpression on ATAD3A protein levels was not readily apparent under these specific conditions. When ME2 was expressed at a higher level, allowing it to more effectively compete for TRIM25 binding, it counteracted the TRIM25-mediated reduction in ATAD3A and mitochondrial proteins (Fig. S8A, B). These findings indicated that, in addition to ATAD3A, there are alternative ways for malic enzyme 2 may inhibit mitophagy. Indeed, among the candidate malic enzyme 2-binding proteins we identified, several may also participate in mitophagy regulation. Thus, malic enzyme 2 likely modulates mitophagy not only via ATAD3A but also through additional interacting proteins. The precise mechanisms through which malic enzyme 2 regulates mitophagy via these other factors remain unclear and requires further investigation.

We further demonstrated that malic enzyme 2 regulates mitophagy through protein-protein interactions independent of catalytic activity. Catalytically inactive malic enzyme 2 mutants retained the ability to suppress mitophagy similarly to wild-type malic enzyme 2 (Fig. 4). This observation parallels a previous report showing that fumarate-induced malic enzyme 2 dimerization promotes mitochondrial biogenesis via DUT activation, an effect dependent on binding rather than enzymatic turnover[17]. Thus, malic enzyme 2 may function as a molecular chaperone in mitochondrial regulation. Metabolomic studies in patients with spondyloarthritis have shown elevated levels of malic acid, succinic acid, and lactate, along with extensive alterations in lipid and amino acid metabolism, indicating marked mitochondrial and metabolic remodeling in vivo [33, 34]. In our model, we found that treatment with malic acid, a substrate of malic enzyme 2, downregulated malic enzyme 2 expression in HepG2 cells but upregulated it in SH-SY5Y cells (Figure S9). In SH-SY5Y cells, we propose that malic acid upregulates ME2 expression to help maintain metabolic balance. In contrast, the hepatoma cell line HepG2 endogenously exhibits high basal ME2 levels, which support elevated reactive oxygen species (ROS) production, a critical factor for hepatoma cell survival and proliferation. To prevent ROS overload, malic acid does not further induce ME2 expression in HepG2 cells; rather, it leads to ME2 downregulation. Our finding that malic enzyme 2 regulates PINK1-Parkin-mediated mitophagy raises the possibility that altered malate/malic enzyme 2 flux in inflammatory diseases such as spondyloarthritis might impact mitochondrial quality control and immune cell function, an intriguing hypothesis that requires future investigation.

## Methods

### Cell culture

293 T (SCSP-502) and HepG2 cell lines were procured from the Cell Bank of the Chinese Academy of Sciences (Shanghai, China). HeLa cells expressing GFP-Parkin and SH-SY5Y cells were generously donated by Dr. Zhiyin Song from Wuhan University, China. All cells were cultured in Dulbecco’s Modified Eagle Medium (Gibco, NY, USA) supplemented with 10% fetal bovine serum (FBS) and 1% penicillin-streptomycin (Gibco) at 37 °C in a humidified incubator with 5% CO₂.

### Plasmids and transfection

shRNA target sequences targeting malic enzyme 2 and ATAD3A were designed using the Wisconsin siRNA Selection Program (https://sirna.wi.mit.edu/). The shRNA sequences employed in this study were provided in Supplementary Table S1.

The target sequences were cloned into the pLKO.1 vector (Addgene #10878). Lentiviral particles were produced by co-transfecting the pLKO.1or shRNA and packaging plasmids psPAX2 and pMD2.G in 293 T cells, with polyethylenimine (PEI) as a transfection reagent. Lentiviral supernatants were harvested at 48- and 72-hours post-transfection and employed to infect the target cells in the presence of 10 μg/mL polybrene. Stable transduction was selected via the addition of 1 mg/ml puromycin 24 hours post-infection.

For the transfection of PINK1 siRNAs, procured from Guangzhou IGE Biotechnology Co., Ltd., the pLKO.1 and malic enzyme 2-knockdown HepG2 cells were transfected with 30 nM of either negative control (NC) siRNA or PINK1 siRNAs utilizing the RNATransMate siRNA transfection reagent (Sangon Biotech (Shanghai) Co., Ltd, E607402). After a 72-hour incubation period post-transfection, the cells were collected for subsequent Western blot analysis or cell viability assessment using the cell counting kit-8 (CCK-8) assay. The specific sequences of the siRNAs employed are detailed in Supplementary Table S1.

### Western blot

Cells were lysed in ice-cold RIPA buffer (ECOTOP, Guangzhou, China) supplemented with 1% protease inhibitor cocktail, 1 mM PMSF, and 2% phosphatase inhibitor cocktail. Following centrifugation at 12,000 × *g* for 10 min at 4 °C, the supernatants were collected as total protein extracts. Protein concentrations were quantified using a BCA protein assay kit (Meilunbio, Dalian, China). Equal quantities of protein were mixed with 5*SDS loading buffer and heated at 95 °C for 10 min. Proteins were then resolved by SDS-polyacrylamide gel electrophoresis (SDS–PAGE) and transferred onto polyvinylidene difluoride (PVDF) membranes. Membranes were blocked with 5% non-fat milk and sequentially incubated with primary antibodies and HRP-conjugated secondary antibodies. Protein bands were detected using enhanced chemiluminescence (ECL). A list of primary antibodies used in this study is provided in Supplementary Table S2. Densitometric analysis of the Western blot bands was conducted using Quantity One software version 4.6.6. Initially, the expression levels of the target proteins were quantified relative to ACTB or GAPDH within the same sample. Subsequently, the relative protein expression levels across different groups were normalized to the respective control group, which was arbitrarily assigned a value of 1. The results are presented as mean ± standard deviation (SD). Statistical significance was evaluated using Student’s *t* test, with *p* values indicated as follows: *p < 0.05; **p < 0.01; ***p < 0.001.

### Co-immunoprecipitation (Co-IP)

48 hours following transfection with overexpression plasmids, cells were lysed in immunoprecipitation (IP) lysis buffer (Beyotime, Shanghai, China) supplemented with a protease inhibitor cocktail, PMSF, and phosphatase inhibitors. Cell lysates were precleared with 20 μL of protein A/G magnetic beads (MCE, NJ, USA) at 4 °C for 4 hours using 1 mg of total protein. The cleared supernatants were transferred to fresh 1.5 mL tubes and incubated overnight at 4 °C with either control immunoglobulin G (IgG) or target-specific antibody, along with 30 μL of protein A/G magnetic beads. Beads were washed three times with washing buffer and resuspended in 60 μL of SDS loading buffer, then boiled at 95 °C for 30 min. The samples were then analyzed by immunoblotting.

### Quantitative real-time PCR (RT–qPCR)

Total RNA was isolated using the RNAiso Plus reagent (TaKaRa, Tokyo, Japan) according to the manufacturer’s instructions. Complementary DNA (cDNA) was synthesized from total RNA using the ABScript II cDNA First Strand Synthesis Kit (ABclonal, Wuhan, China). Quantitative PCR was conducted using SYBR Green Master Mix (ABclonal). Gene expression levels were normalized to the housekeeping gene ACTB using the 2^–ΔΔCt^ method. The primer sequences employed in this study are detailed in Supplementary Table S3.

### Immunofluorescence (IF) analysis

The Cells were seeded onto coverslips at a suitable density. After washing with PBS, cells were fixed with 4% paraformaldehyde for 15 minutes and then permeabilized with 0.2% Triton X-100 for 10 minutes and blocked with 5% bovine serum albumin (BSA) for 1 hour at room temperature. Subsequently, cells were then incubated with primary antibodies overnight at 4 °C, followed by incubation with fluorophore-conjugated secondary antibodies for 2 hours at room temperature in the dark. Nuclei were counterstained with DAPI for 15 minutes. Finally, cells were imaged using a Leica TCS SP8 confocal microscope.

### Cell viability and colony formation assays

Cell viability was evaluated using the Cell Counting Kit-8 (CCK-8, Meilunbio, Dalian, China). CCK-8 reagent was added to 96-well plates seeded with HepG2 cells and incubated at 37 °C in the dark for 2 hours, and absorbance was measured at 450 nm with a microplate reader. For colony formation assays, 2000 cells were seeded per well in 6-well plates and cultured for two weeks, with medium replacement every three days. After colony formation, cells were fixed and stained with crystal violet.

### Oxygen consumption rate (OCR) measurement

The oxygen consumption rate (OCR) was measured using the Seahorse XF Analyzer (Agilent Technologies, CA, USA). The knockdown or overexpression malic enzyme 2 HepG2 cells were seeded at 2.0 × 10⁴ cells per well in Seahorse XF 96-well culture plates and incubated overnight. The culture medium was replaced with Seahorse XF Base Medium (pH 7.4) supplemented with 25 mM glucose, 4 mM L-glutamine, and 2 mM sodium pyruvate, and incubated in a non-CO₂ incubator at 37 °C for 1 hour before the assay. Cells were sequentially treated with 1 μM oligomycin, 1 μM FCCP, and 2.5 μM antimycin A/rotenone to evaluate mitochondrial respiration. OCR values were normalized to total protein content and analyzed using WAVE software.

### 3D spheroid formation assay

HepG2 cells were seeded into ultra-low attachment 12-well plates. The wells were pre-coated with Matrigel (YEASEN, Shanghai, China), allowed to solidify at 37 °C, and then overlaid with 1000 cells mixed with Matrigel. After 4 days of incubation in a 5% CO₂ humidified incubator, spheroid formation was imaged and quantified under a microscope.

### Subcellular fractionation

HepG2 cells cultured in 6-cm dishes were collected and centrifuged at 600 × *g* for 5 min. The cell pellets were resuspended in digitonin lysis buffer (250 μg/mL digitonin supplemented with phosphatase inhibitors, PMSF, and protease inhibitors) and incubated at room temperature for 10 min. Subsequently, the lysates were centrifuged at 10,000 × *g* for 10 minutes at 4 °C to isolate the supernatant, which was designated as the cytosolic fraction (Cyto). The reserved pellet was resuspended in mitochondrial lysis buffer (10 mM Tris-HCl, 150 mM NaCl, 2 mM EDTA, 0.2% Triton X-100, and 0.3% NP-40, supplemented with protease inhibitors) and incubated on ice for 30 minutes. Following centrifugation at 12,000 × *g* for 30 min at 4 °C, the supernatant was collected and designated as the mitochondrial fraction (Mito).

### Data analysis

Statistical analyses were conducted using GraphPad Prism 8.0 software. Data are presented as mean ± standard deviation (SD). Statistical significance was evaluated using Student’s *t* test, with *p* values indicated as follows: *p < 0.05; **p < 0.01; ***p < 0.001. All experiments were independently repeated at least three times.

## Conclusion

Collectively, our study reveals a previously unrecognized role of malic enzyme 2 in maintaining mitochondrial quality control by modulating the TRIM25-ATAD3A-PINK1 axis. By preventing ATAD3A degradation, ME2 suppresses PINK1-Parkin-mediated mitophagy, preserves mitochondrial integrity, and supports proliferation in hepatocellular carcinoma cells. These findings expand the function of malic enzyme 2 beyond its classical metabolic roles and highlight the interplay between mitochondrial metabolism and quality control in cell biology.

## Supplementary information

Supplementary figures

Supplementary tables

## Data Availability

All data supporting the findings of this study are included in the main text, supplementary information, and supplemental material files. Original Western blot images and quantitative data are provided as Supplemental Material. The data that support this study are available on request from the corresponding author upon reasonable request. Original western blot images are provided as Supplemental Material titled “Original Data”.
