# Supplementary material for: Malic enzyme 2 suppresses PINK1-Parkin-mediated mitophagy by stabilizing ATAD3A via competitive interaction with TRIM25
Source: Cell Death Dis. 2026 Mar 24;17(1):353. doi: 10.1038/s41419-026-08623-2 (PMC13039799; doi:10.1038/s41419-026-08623-2)
Supplement: Supplementary file 1 — Supplementary figures [file 41419_2026_8623_MOESM1_ESM.docx]

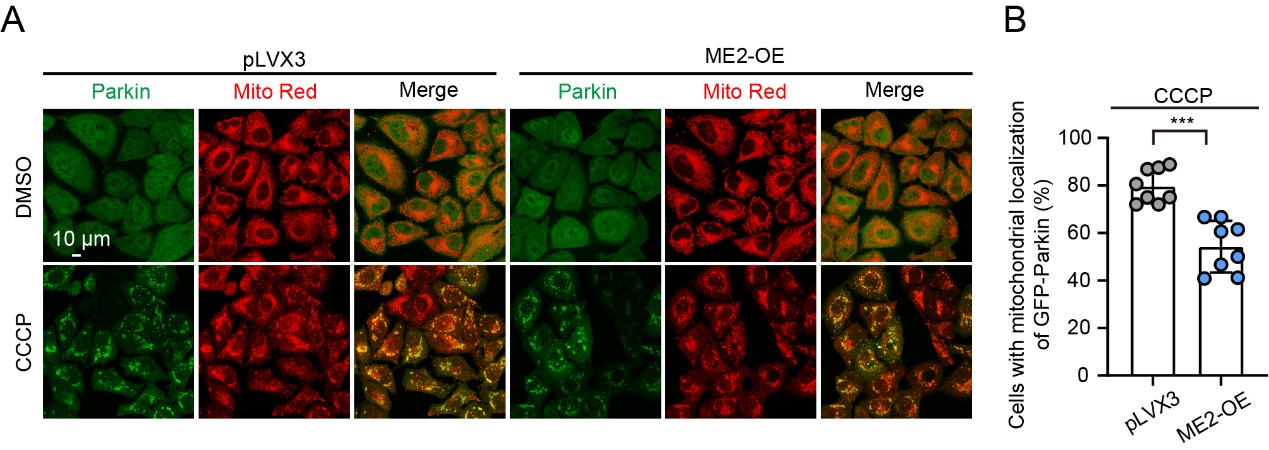


**Supplementary figure 1. Malic enzyme 2 (ME2) overexpression suppresses mitophagy. A-B.** Representative images (A) and quantification (B) of Parkin and MitoTracker Red colocalization in control and ME2-overexpressed HepG2 cells following treatment with 10 μM CCCP for 6 hours. Scale bar, 10 μm. *n* = 8. *** p < 0.001.


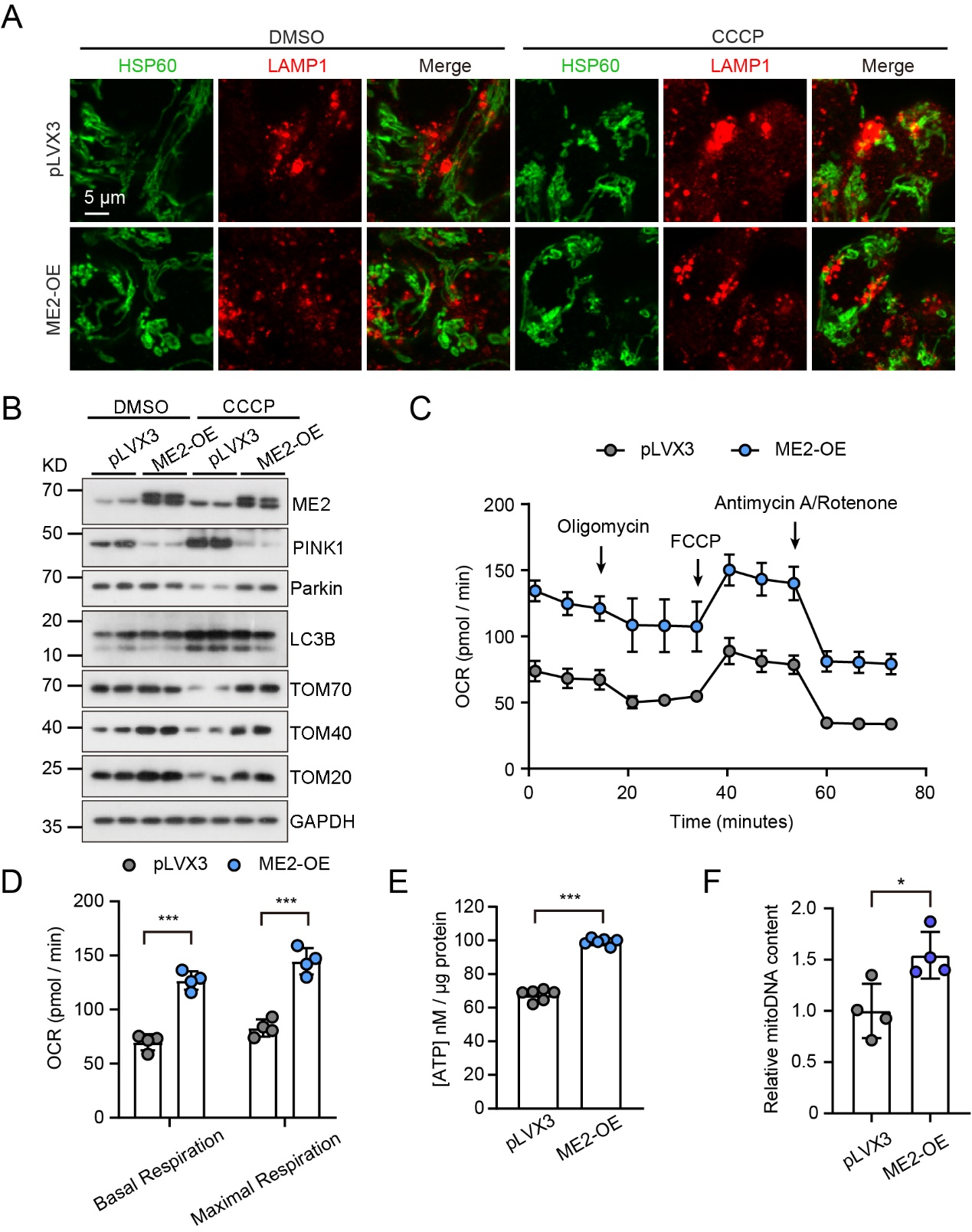


**Supplementary figure 2. Malic enzyme 2 (ME2) overexpression represses mitophagy and improves mitochondrial function. A.** Representative images showing LAMP1 (lysosomal marker) and HSP60 colocalization in control and ME2-overexpressed HepG2 cells following treatment with 10 μM CCCP for 6 hours. Scale bar, 5 μm. **B.** Western blot analysis of mitophagy-related proteins in control and ME2-overexpressed HepG2 cells. **C-D.** Oxygen consumption rate (OCR) analysis in control and ME2-knockdown HepG2 cells. *n* = 4. **E.** Quantification of ATP levels. *n* = 6. **F.** Quantification of mitochondrial DNA copy numbers. *n* = 4. * p < 0.05, *** p < 0.001.


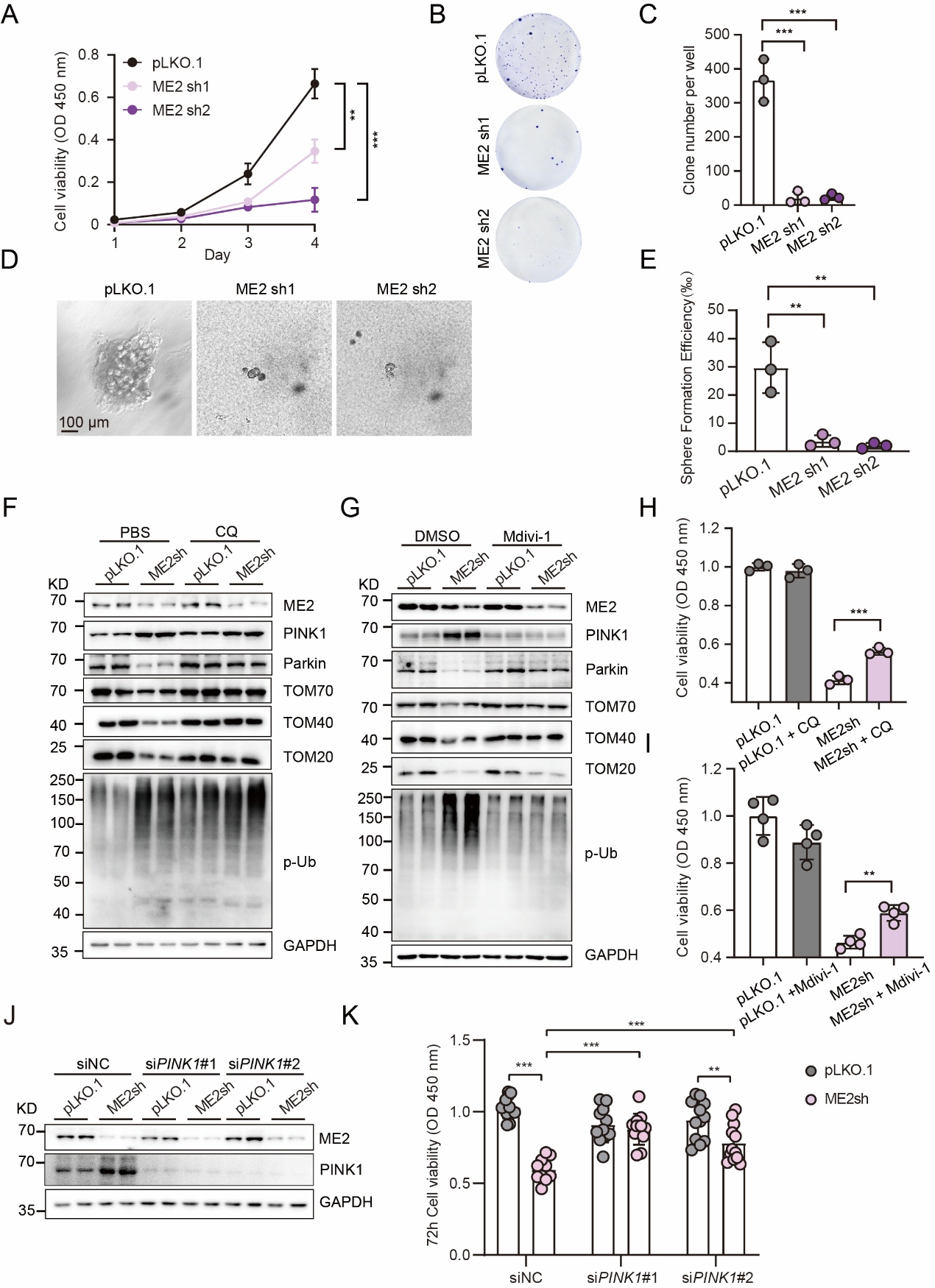


**Supplementary figure 3. Malic enzyme 2 (ME2) deficiency represses hepatoma cancer cell proliferation via hyperactivated mitophagy. A.** Cell viability measured by CCK-8 assay in pLKO.1 and ME2 knockdown HepG2 cells. *n* = 3. **B-C.** Representative images (B) and quantification (C) of colony formation assay in pLKO.1 and ME2 knockdown HepG2 cells. *n* = 3. **D-E.** Representative images (D) and quantification (E) of tumor-sphere formation in pLKO.1 and ME2 knockdown HepG2 cells. *n* = 3. **F.** Western blot analysis of mitophagy-related proteins in control and ME2-knockdown HepG2 cells treatment with 50 μM chloroquine (CQ) for 24 hours. **G.** Cell viability measured by CCK-8 assay in pLKO.1 and ME2 knockdown HepG2 cells following treatment with 10 μM chloroquine (CQ) for 72 hours. *n =* 3. **H.** Western blot analysis of mitophagy-related proteins in control and ME2-knockdown HepG2 cells treatment with 50 μM Mdivi-1 for 24 hours. **I.** Cell viability measured by CCK-8 assay in pLKO.1 and ME2 knockdown HepG2 cells following treatment with 2.5 μM Mdivi-1 for 72 hours. *n =* 4*.* **J.** Western blot analysis of ME2 and PINK1. pLKO.1 and ME2 knockdown HepG2 cells were transfected with PINK1 siRNAs for 72 hours. **K.** Cell viability measured by CCK-8 assay in pLKO.1 and ME2 knockdown HepG2 cells. Cells were transfected with PINK1 siRNAs for 72 hours. *n=* 10*.* ** p < 0.01, *** p < 0.001.


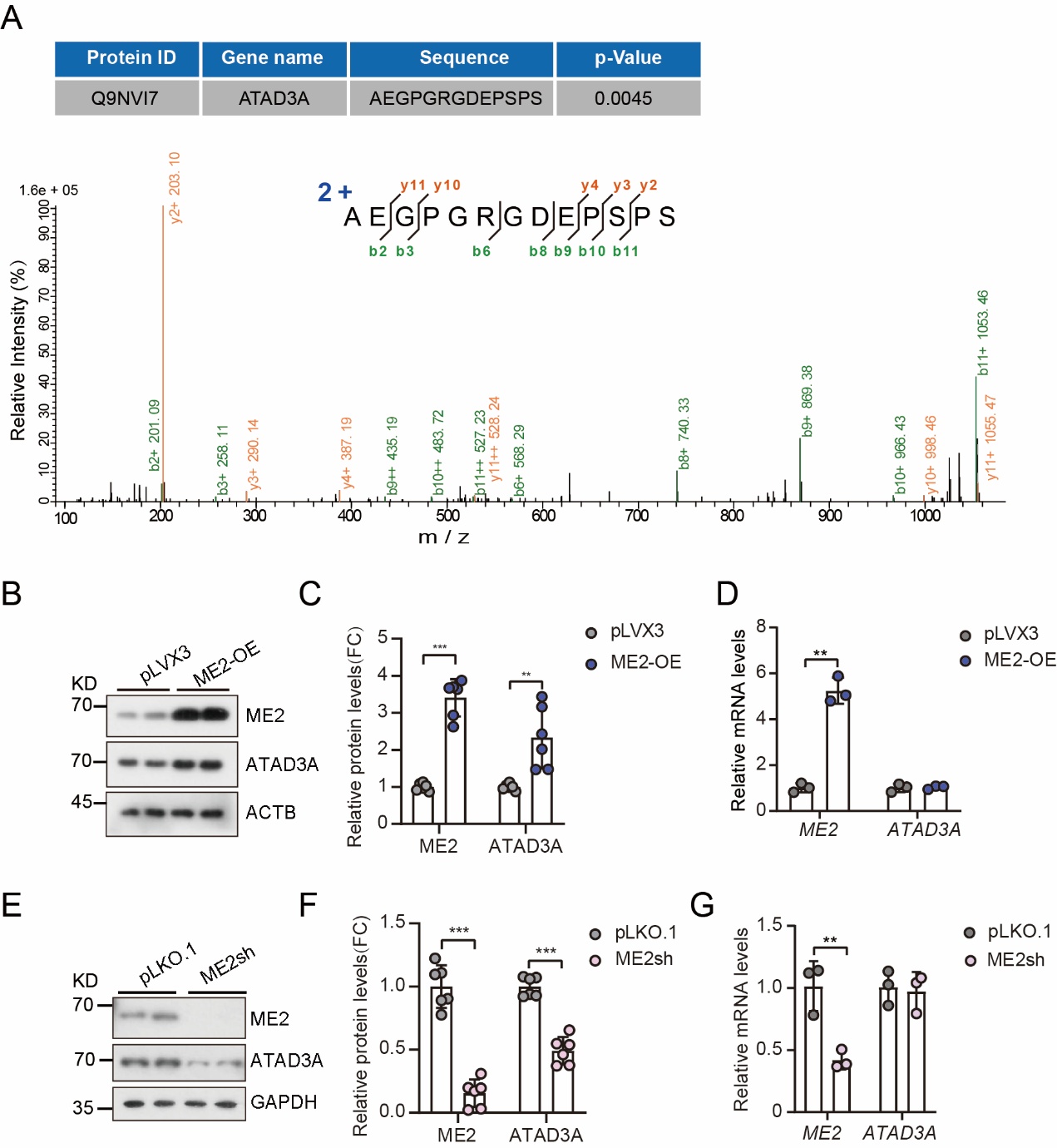


**Supplementary figure 4. Malic Enzyme 2 (ME2) interacts with and regulates the expression of ATAT3A at the protein level. A.** ATAD3A was identified as a ME2-interacting protein through Co-immunoprecipitation (Co-IP) followed by LC–MS analysis in 293T cells. **B-C.** Western blot (B) and quantification (C) of ATAD3A expression in ME2 knockdown HepG2 cells. **D.** qPCR analysis of ATAD3A expression in ME2 knockdown HepG2 cells. *n* = 3. **E-F.** Western blot (E) and quantification (F) analysis of ATAD3A expression in ME2-overexpressed HepG2 cells. **G.** qPCR analysis of ATAD3A expression in ME2 ME2-overexpressed HepG2 cells. *n* = 3. ** p < 0.01, *** p < 0.001.


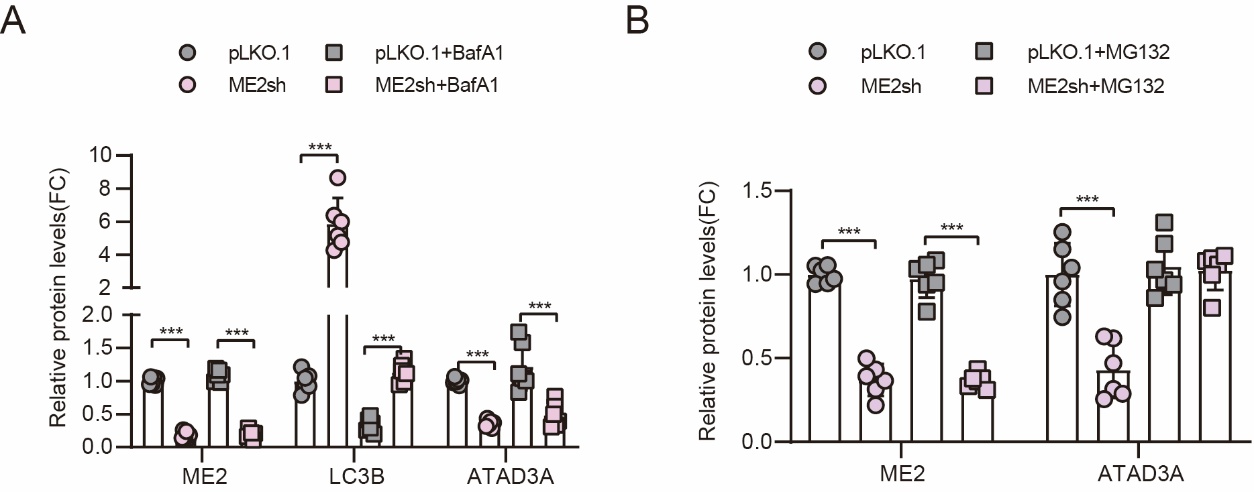


**Supplementary figure 5. Malic enzyme 2 (ME2) regulates ATAD3A expression via the proteasomal degradation. A-B.** Quantification results of western blots in figure 5 B (A) and figure 5C (B). pLKO.1 and ME2 knockdown HepG2 cells were treated with 100 nM BafA1 (A) or 10 μM MG132 (B) treatment for 8 hours. *n* = 6. *** p < 0.001.


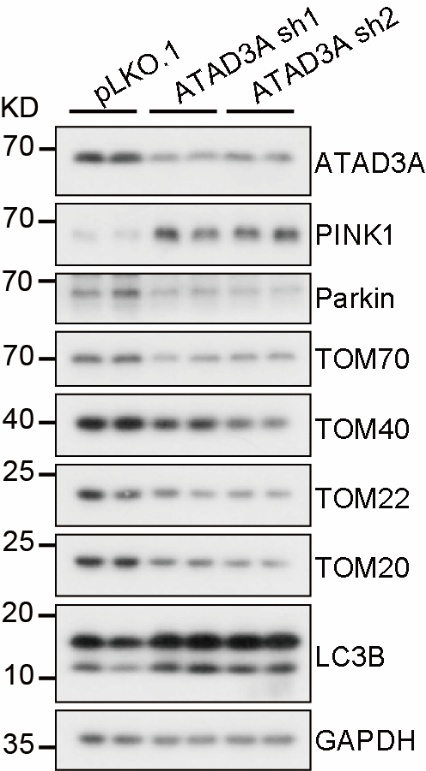


**Supplementary figure 6. Knockdown of ATAD3A represses mitophagy. proteasomal degradation.** Representative western blots of the indicated mitophagy-related proteins in pLKO.1 and ATAD3A knockdown HepG2 cells.


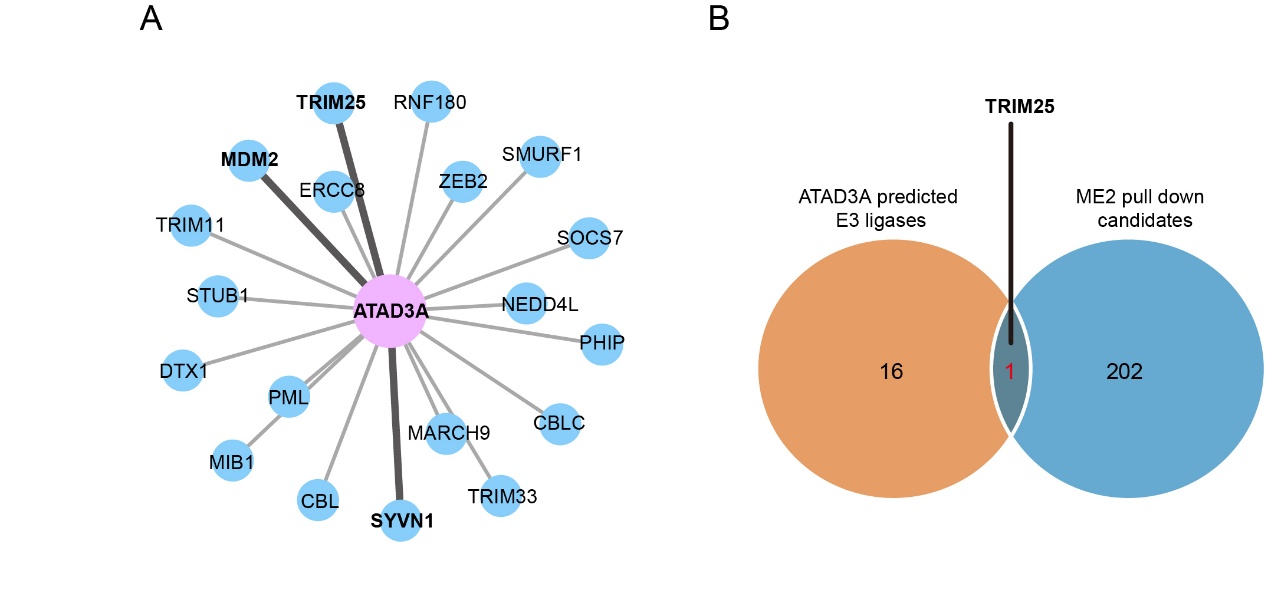


**Supplementary figure 7. Bioinformatic prediction of ME2-regulated E3 ligases targeting ATAD3A ubiquitination. A.** Predicted E3 ubiquitin ligases of ATAD3A identified using the Ubibrowser database. **B.** Venn diagram showing the overlap among predicted ATAD3A E3 ligases and ME2-interacting proteins (pulldown candidates).


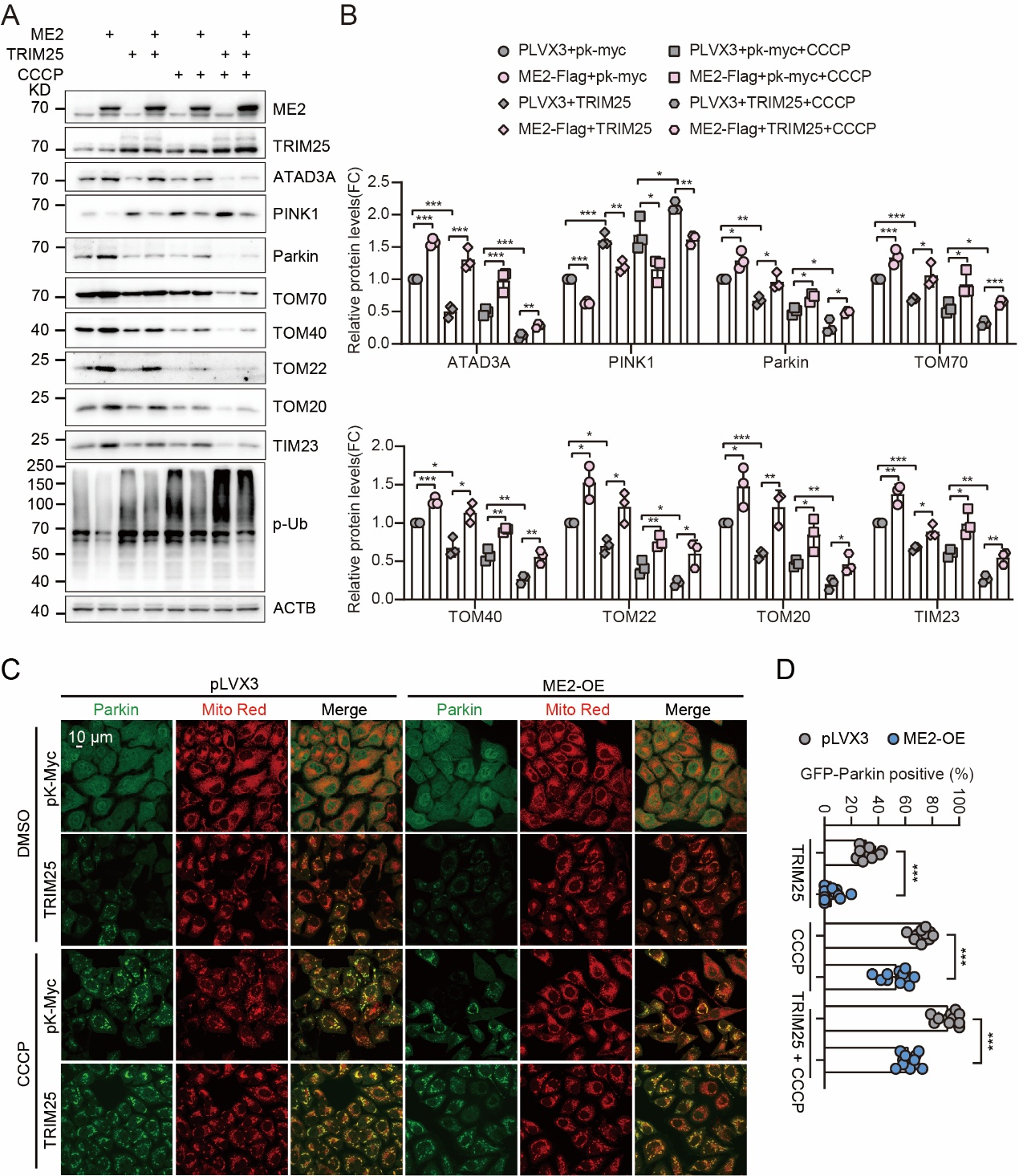


**Supplementary figure 8. Overexpression of malic enzyme 2 (ME2) attenuates TRIM25-mediated mitophagy. A-B.** Western blot analysis (A) and quantification (B) of mitophagy-associated proteins. The control and ME2-overexpressed HepG2 cells were transfected with pK-Myc or TRIM25 for 48 hours and then treated with 10 μM CCCP for another 6 hours. *n =* 3. **C-D.** Representative images (C) and quantification (D) of Parkin and mitochondria colocalization in control and ME2-overexpressed HepG2 cells following ectopic expression of TRIM25 with or without 10 μM CCCP treatment for 6 hours. Scale bar, 10 μm. *n* = 10. * p < 0.05, ** p < 0.01, *** p < 0.001.


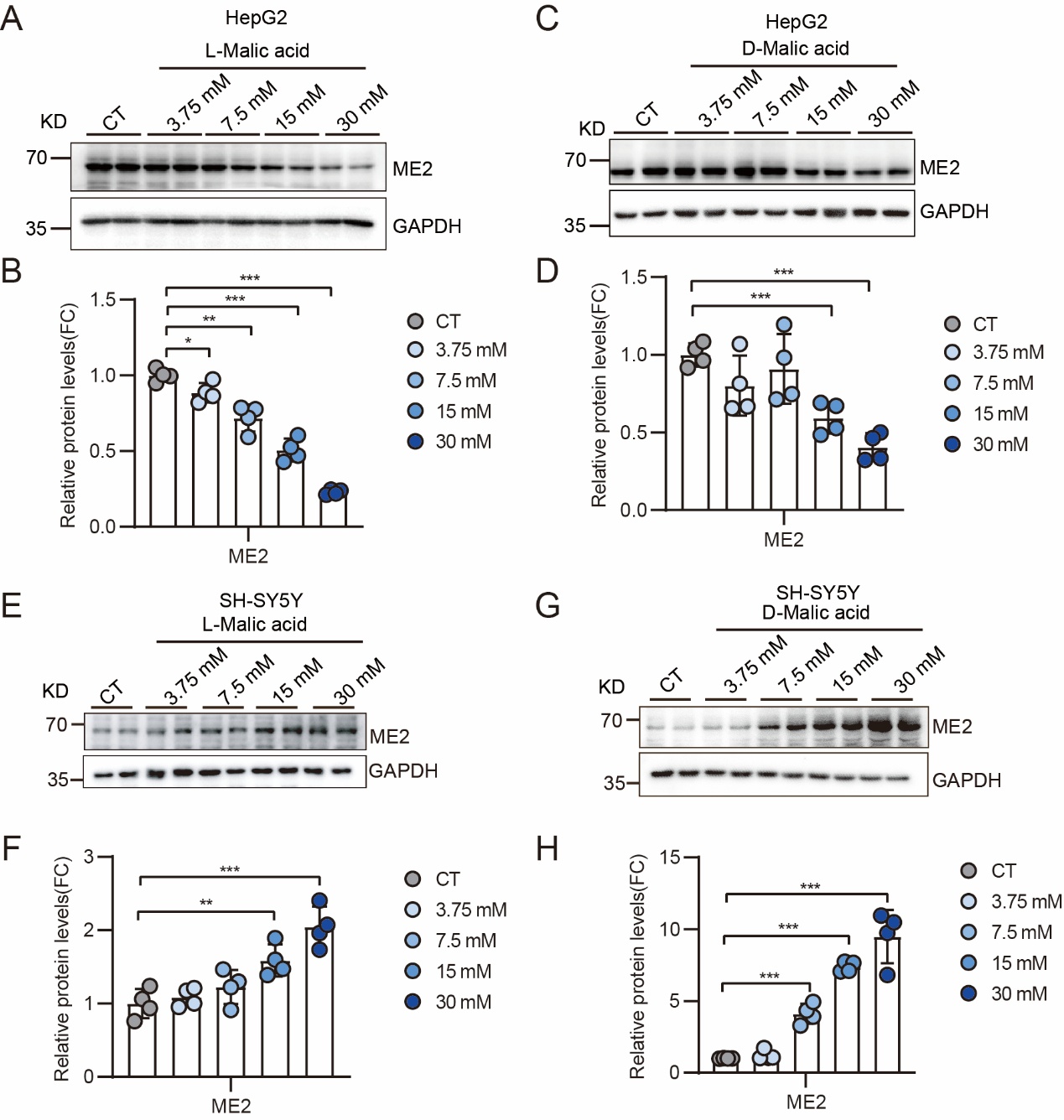


**Supplementary figure 9. Malic acid regulates the expression of malic enzyme 2 (ME2) in HepG2 and SH-SY5Y cells. A-D.** Western blot (A, C) and quantification (B, D) of ME2 protein level in HepG2 cells following treatment with the indicated concentration of L-malic acid and D-malic acid for 24 hours. **E-H.** Western blot (E, G) and quantification (F, H) of ME2 protein level in SH-SY5Y cells following treatment with the indicated concentration of L-malic acid and D-malic acid for 24 hours. *n* = 4. * p < 0.05, ** p < 0.01, *** p < 0.001.
