# Supplementary material for: Malic enzyme 2 suppresses PINK1-Parkin-mediated mitophagy by stabilizing ATAD3A via competitive interaction with TRIM25
Source: Cell Death Dis. 2026 Mar 24;17(1):353. doi: 10.1038/s41419-026-08623-2 (PMC13039799; doi:10.1038/s41419-026-08623-2)
Supplement: Supplementary file 2 — Supplementary tables [file 41419_2026_8623_MOESM2_ESM.docx]

**Table S1. Gene-shRNA and siRNA sequence**

| Target Gene | Sequence (5’-3’) |
| --- | --- |
| ME2  (shRNA-1) | F:CCGGCGAAAGCTATTACTGACAGATACTCGAGTATCTGTCAGTAATAGCTTTCTTTTTG  R:AATTCAAAAATATCTGTCAGTAATAGCTTTCCTCGAGGAAAGCTATTACTGACAGATAG |
| ME2  (shRNA-2) | F:CCGGCTTTTTGCGGCATATTAGTGACAGTGTTCTCGAGAACACTGTCACTAATATGCCG  R:AATTCAAAAAAACACTGTCACTAATATGCCGCTCGAGCGGCATATTAGTGACAGTGTTG |
| ATAD3A  (shRNA-1) | F:CCGGCCTGCACATTTAGGATATGCTCTCGAGAGCATATCCTAAATGTGCAGGTTTTTG  R:AATTCAAAAACCTGCACATTTAGGATATGCTCTCGAGAGCATATCCTAAATGTGCAGG |
| ATAD3A  (shRNA-2) | F:CCGGCATAGCAACAAGGAACACCAACTCGAGTTGGTGTTCCTTGTTGCTATGTTTTTG  R:AATTCAAAAACATAGCAACAAGGAACACCAACTCGAGTTGGTGTTCCTTGTTGCTATG |
| PINK1  (siRNA-1) | sense: CCAUCAAGAUGAUGUGGAAtt  antisense: UUCCACAUCAUCUUGAUGGtt |
| PINK1  (siRNA-2) | sense: GGAGCAGUCACUUACAGAAtt  antisense: UUCUGUAAGUGACUGCUCCtt |

**Table S2. Primary and secondary antibodies used in the present study**

| **Antibody (Application)** | **Company** | **Catalog number** |
| --- | --- | --- |
| ME2 (WB / IP) | Proteintech | 24944-1-AP |
| GAPDH (WB) | Cell Signaling Technology | #2118 |
| Parkin (WB) | Proteintech | 14060-1-AP |
| PINK1(WB) | Proteintech | 23274-1-AP |
| SQSTM1/p62 (WB) | Sigma | P0067 |
| LC3B (WB / IF) | Sigma | L7543 |
| TOM70(WB) | Proteintech | 14528-1-AP |
| TOM40(WB) | Proteintech | 18409-1-AP |
| TOM22(WB) | Proteintech | 11278-1-AP |
| TOM20 (WB / IF) | Proteintech | 11802-1-AP |
| TIM23(WB) | Proteintech | 11123-1-AP |
| HSP60(IF) | Proteintech | 15282-1-AP |
| LAMP1(IF) | Cell Signaling Technology | #9091 |
| ATAD3A(WB / IP) | NOVUS | H00055210-D01P |
| ACTB (WB) | Sigma | A1978 |
| P-Ubiquitin(WB) | Cell Signaling Technology | #62802 |
| Ubiquitin (WB) | Proteintech | 10201-2-AP |
| TRIM25(WB / IP) | Proteintech | 12573-1-AP |
| Flag (WB / IP) | Sigma | F1804 |
| Myc (IP) | Proteintech | 60003-2-Ig |
| HA-tag(IP) | Proteintech | 66006-2-IG |
| Mouse Anti-Rabbit IgG (Light-Chain Specific) (D4W3E) mAb (HRP Conjugate) | Cell Signaling Technology | 93702S |
| VeriBlot for IP Detection Reagent (HRP) | Abcam | ab131366 |
| Peroxidase-AffiniPure Goat Anti-Mouse IgG (H+L) | Jackson ImmunoResearch | 115-035-003 |
| Peroxidase-AffiniPure Goat Anti-Rabbit IgG (H+L) | Jackson ImmunoResearch | 111-035-003 |
| Goat anti-Mouse IgG(H+L) Cross-Adsorbed Secondary Antibody, Alexa Fluor 488 | Thermo | A28175 |
| Alexa Fluor® 488-AffiniPure Goat Anti-Rabbit IgG (H+L) | Jackson ImmunoResearch | 111-545-144 |
| Alexa Fluor® 594-AffiniPure Goat Anti-Mouse IgG (H+L) | Jackson ImmunoResearch | 115-585-146 |

**Table S3. Primers sequence in the present study**

| **Gene** | **Sequences: (5’-3’)** |
| --- | --- |
| *ME2-Human* | F:ATCCTACAGCACAGGCAGAGTG |
|  | R:TGACCTGGTGTAAAGACTCGCC |
| *ATAD3A-Human* | F:CGCCATAGCAACAAGGAACACC |
|  | R:ATGGCGTAGTCCATGCCTGAGT |
| *ACTB*-Human | F:CATGTACGTTGCTATCCAGGC |
|  | R:CTCCTTAATGTCACGCACGAT |
| *UUR*-Human | F:CACCCAAGAACAGGGTTTGT |
|  | R:TGGCCATGG GTATGTTGTTA- |
| *beta-2-Microglobulin-*Human | F:TGCTGTCTCCATGTTTGATGTATCT |
|  | R:TCTCTGCTCCCCACCTCTAAGT |

**Table S4. Reagents and plasmids used in the present study**

| **Chemicals or plasmids** | **Company** | **Catalog number** |
| --- | --- | --- |
| ABScript II cDNA First Strand Synthesis Kit | ABclonal | RK20400 |
| Bafilomycin A | MCE | 88899-55-2 |
| CCCP | MCE | HY-100941 |
| Cell lysis buffer for Western and IP | Beyotime | P0013J |
| Chloroquine | MCE | HY-17589AR |
| DAPI | Sigma | D8417 |
| DMEM | Gibco | C11995500BT |
| DMSO | Biosharp | BL165A |
| FBS | Sigma | 1943609-65-1 |
| Hank's 1× Balanced Salt Solutions | HyClone | SH30268.01 |
| Mito-Tracker Red | Beyotime | C1035 |
| Mdivi-1 | MCE | HY-15886 |
| MG-132 | TargetMol | 133407-82-6 |
| Opti-MEM (1X) | Gibco | 31985070 |
| Penicillin and streptomycin | Gibco | 15410-122 |
| Polybrene | Sigma | 28728-55-4 |
| Protein A/G Magnetic Beads | MCE | HY-K0202 |
| Puromycin | Amresco | J593 |
| RIPA Lysis Buffer | ECOTOP | ES-8148 |
| Mitochondrial Membrane Potential Assay Kit with TMRE | Beyotime | C2001S |
| MitoSOX™ Red | Beyotime | S0061M |
| Crystal violet | Aladdin | C110702-100g |
| Enhanced ATP Assay Kit | Beyotime | S0027 |
| MYC Tag protein immunoprecipitation kit | AlpalifeBio | #KTSM1365 |
| Flag Tag protein immunoprecipitation kit | AlpalifeBio | #KTSM1361 |
| Dimethyl L-(-)-Malate | Aladdin | D102009 |
| Dimethyl D-Malate | Aladdin | D103127 |
| **Plasmids** |  |  |
| pLKO.1 puro | Addgene | #8453 |
| pLV3-CMV-ME2(human)-3×FLAG-CopGFP-Puro | Miaoling plasmid | [P43466](https://www.miaolingbio.com/plasmid/P43466) |
| pLV3-CMV-ATAD3A(human)-3×FLAG-CopGFP-Puro | Miaoling plasmid | P62268 |
| pLV2-CMV-3×Myc-TRIM25(human)-Puro | Miaoling plasmid | P52796 |
| [pLV3-CMV-COX8-EGFP-mCherry-Puro](https://www.miaolingbio.com/plasmid/P57619) | Miaoling plasmid | [P57619](https://www.miaolingbio.com/plasmid/P57619) |
| pLV3-CMV-ME2-R67Q-3×FLAG-CopGFP-Puro | This paper | Expression constructed generated in the lab |
| pLV3-CMV-ME2-Y112A-K183A-3×FLAG-CopGFP-Puro | This paper | Expression constructed generated in the lab |
| pK-Myc | Addgene | #19400 |
| pRK5-HA-Ubiquitin-WT | Addgene | #17608 |
